# Preserving the Immune‐Privileged Niche of the Nucleus Pulposus: Safeguarding Intervertebral Discs from Degeneration after Discectomy with Synthetic Mucin Hydrogel Injection

**DOI:** 10.1002/advs.202404496

**Published:** 2024-08-29

**Authors:** Huan Wang, Song Chen, Zhao Liu, Qingchen Meng, Rita Sobreiro‐Almeida, Ling Liu, Håvard Jostein Haugen, Jiaying Li, João F. Mano, Youzhi Hong, Thomas Crouzier, Hongji Yan, Bin Li

**Affiliations:** ^1^ Medical 3D Printing Center Orthopedic Institute Department of Orthopedic Surgery The First Affiliated Hospital School of Basic Medical Sciences Suzhou Medical College Soochow University Suzhou Jiangsu 215000 China; ^2^ National University of Singapore Suzhou Research Institute Suzhou Jiangsu 215000 China; ^3^ CICECO‐Aveiro Institute of Materials Department of Chemistry University of Aveiro Aveiro 3810‐193 Portugal; ^4^ Department of Biomaterials Institute for Clinical Dentistry University of Oslo PO Box 1109 Blindern Oslo 0376 Norway; ^5^ Department of Health Technology DTU Ørsteds Plads, building 345C DK‐2800 Kgs, Lyngby Copenhagen Denmark; ^6^ Department of Medical Cell Biology Uppsala University Uppsala 75123 Sweden; ^7^ AIMES – Center for the Advancement of Integrated Medical and Engineering Sciences at Karolinska Institute and KTH Royal Institute of Technology Stockholm 17177 Sweden; ^8^ MOE Key Laboratory of Geriatric Diseases and Immunology Suzhou Medical College Soochow University Suzhou Jiangsu 215000 China; ^9^ Collaborative Innovation Center of Hematology Soochow University Suzhou Jiangsu 215000 China

**Keywords:** immune‐privileged niche, intervertebral disc degeneration, macrophage, mucin gel, prevention

## Abstract

Intervertebral disc (IVD) herniation is a prevalent spinal disorder, often necessitating surgical intervention such as microdiscectomy for symptomatic relief and nerve decompression. IVDs comprise a gel‐like nucleus pulposus (NP) encased by an annulus fibrosus (AF), and their avascular nature renders them immune‐privileged. Microdiscectomy exposes the residual NP to the immune system, precipitating an immune cell infiltration and attack that exacerbates IVD degeneration. While many efforts in the tissue engineering field are directed toward IVD regeneration, the inherently limited regenerative capacity due to the avascular and low‐cellularity nature of the disc and the challenging mechanical environment of the spine often impedes success. This study, aiming to prevent IVD degeneration post‐microdiscectomy, utilizes mucin‐derived gels (Muc‐gels) that form a gel at the surgical site, inspired by the natural mucin coating on living organisms to evade immune reorganization. It is shown that type I macrophages are present in severely degenerated human discs. Encapsulating IVDs within Muc‐gels prevents fibrous encapsulation and macrophage infiltration in a mouse subcutaneous model. The injection of Muc‐gels prevents IVD degeneration in a rat tail IVD degeneration model up to 24 weeks post‐operation. Mechanistic investigations indicate that Muc‐gels attenuate immune cell infiltration into NPs, offering durable protection against immune attack post‐microdiscectomy.

## Graphical abstract

This study unequivocally establishes the multifaceted utility of mucin hydrogels (Muc‐gels), showcasing their efficacy as both a physical shielding barrier and an immune‐isolating barrier for nucleus pulposus. This novel dual‐functionality presents a promising avenue for mitigating the recurrence rate of discectomy and impeding the progression of intervertebral disc degeneration.

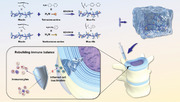

## 1 Introduction

Cell bypassing immune recognition through coatings of biopolymers such as mucins and mucin‐like glycoproteins is a good example how nature can offer valuable insights for biomaterial design. Mucins, a class of glycoproteins, have recently emerged as a fascinating area of research in biomedical applications, particularly due to their pivotal role in the mammalian immune regulatory system.^[^ ^1^ ^]^ These glycoproteins are the key components of mucus, a viscoelastic gel‐like substance that serves as a protective barrier,^[^ ^2^ ^]^ providing hydration, lubrication, and defense to various epithelial surfaces. Mucins exhibit multifunctional properties, characterized by densely glycosylated serine and threonine‐rich regions terminated by various sugars, such as sialic acids and fucose residues, conferring unique immunological characteristics.^[^ ^3^ ^]^ Mucins are also strongly immunomodulatory. For instance, intestinal MUC2 mucins dampen the inflammatory activation of dendritic cells, leading to increased secretion of the anti‐inflammatory cytokine IL‐10. Conversely, contradictory findings suggest that these mucins may also enhance the expression of the proinflammatory cytokine IL‐8 in dendritic cells.^[^ ^4^ ^]^ Moreover, in the context of cancer, altered glycosylation patterns of membrane‐bound MUC1 mucins, coupled with substantial mucin secretion, contribute to immune evasion and establish a physical and biochemical protective barrier for tumors to safeguard the tumor by concealing antigens and regulating the activity of macrophages^[^ ^5^ ^]^ and NK cells.^[^ ^6^ ^]^ Further, trypanosoma cruzi parasites *(T. cruzi)* transmitted to humans by triatomine insects produce mucins not only obstruct antigen recognition but also actively modulate the immune response by inhibiting activation and production of pro‐inflammatory cytokines in macrophages^[^ ^7^ ^]^ and T cells,^[^ ^8^ ^]^ thereby ensuring their survival within the host.^[^ ^9^ ^]^

Inspired by the immunomodulatory properties of mucins, our research endeavors have focused on developing synthetic mucin hydrogels.^[^ ^10^ ^]^ These hydrogels have demonstrated remarkable capabilities in suppressing immune cell activation upon viral exposure, acting as potent defence mechanisms against infection.^[^ ^11^ ^]^ Additionally, mucin hydrogels can inhibit immune cell recruitment and activation, effectively evading fibrotic isolation,^[^ ^12^ ^]^ and can suppress the activation of the human complement system, a critical component of the immune response.^[^ ^13^ ^]^ Remarkably, these hydrogels have also shown to induce macrophage polarization toward the M2 phenotype, promoting healing and revascularization in critical size calvarial bone defects in rats.^[^ ^14^ ^]^

Intervertebral disc (IVD)herniation is a prevalent spinal condition characterized by considerable pain and disability,^[^ ^15^ ^]^ often requiring surgical interventions for symptom relief and decompression of affected spinal nerves.^[^ ^16^ ^]^ The procedures typically involve the removal of the herniated disc, employing techniques like microdiscectomy or minimally invasive surgery. The IVD comprises a gel‐like substance called the nucleus pulposus (NP), encased by the annulus fibrosus (AF).^[^ ^17^ ^]^ The NP, characterized by its immune‐privileged nature, exhibits limited immune cell infiltration and immune responses compared to other tissues.^[^ ^18^ ^]^ This immune privilege arises from the disc's avascular nature and the presence of immunomodulatory molecules.^[^ ^19^ ^]^ However, surgical removal of a herniated disc exposes the NP residues to the immune system, triggering immune cells, such as macrophages and lymphocytes, to recognize it as foreign or damaged material. Consequently, immune attack, accompanied by the release of enzymes, inflammatory cytokines and reactive oxygen species (ROS). Increasing evidence have confirmed that numerous inflammatory factors (e.g., interleukin (IL)−1β, tumor necrosis factor (TNF)‐α) found in degenerated IVD tissue are products of macrophages.^[^ ^20^ ^]^ These factors can cause extracellular matrix degradation and increased pain sensitivity.^[^ ^21^ ^]^ Additionally, they can induce an inflammatory response in NP cells, thereby amplifying the inflammatory damage and leading to NP deterioration, disruption of nutrient supply, and exacerbated degeneration.^[^ ^22^ ^]^ These suggest that macrophages could regulate immunological responses involved in IVD degeneration. However, these surgical procedures do not address the underlying disease, leading to further degeneration and potential re‐herniation, which still occurs in ≈5%–25% of discectomy cases.^[^ ^23^ ^]^

Conservative treatments following disc herniation surgery, including anti‐inflammatory medications^[^ ^24^ ^]^ and localized corticosteroid injections,^[^ ^25^ ^]^ provide immediate relief by mitigating pain, swelling, and inflammation. Physical therapy and rehabilitation exercises are also employed to restore function and promote tissue recovery.^[^ ^26^ ^]^ Unfortunately, these treatments often fail to fully resolve the underlying structural issues or prevent future herniation recurrence. To address these challenges, ongoing research in IVD biology and tissue engineering aims to develop strategies that mitigate immune responses and facilitate NP regeneration. Promising approaches involve the use of materials with anti‐inflammatory^[^ ^27^ ^]^ or anti‐ROS^[^ ^28^ ^]^ functions, which regulate the immune response and foster tissue repair. However, the avascular and low‐cellularity nature of the disc and the challenging mechanical environment of the spine pose significant hurdles for implementing these strategies. The preservation of the immune‐privileged niche following discectomy is an area that lacks specific studies, despite its potential to significantly enhance patient outcomes and reduce the risk of recurrent disc herniation.

**Scheme 1:**
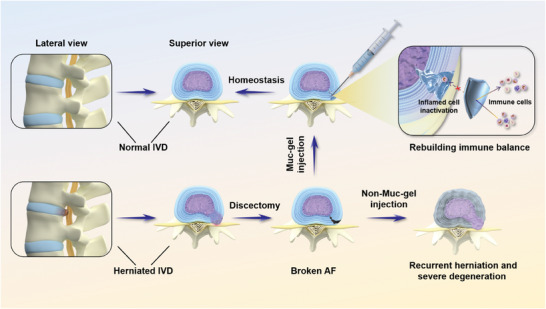
The in situ gelling Muc‐gels intervention following discectomy prevent IVD degeneration. The introduction of “clickable” functionality onto mucins are achieved by reacting amine‐tetrazine (Tz) or amine‐norbornene (Nb) with activated carboxylic groups found in mucins. When Muc‐Tz and Muc‐Nb are mixed in a solution, robust covalent bonds are formed via an inverse electron‐demand Diels‐Alder cycloaddition reaction, leading to formation of Muc‐gels within 3 to 5 min. Thus, these gels can further solidify in situ upon injection into the surgical site in a rat tail IVD degeneration model. This study demonstrated that injecting mucin‐gels at the surgical site following discectomy formed a physical and immune barrier to effectively prevent NP herniation and IVD degeneration, as evidenced by the sustained normal relative water content in the NP, disc height index, and integrity, along with the observation of biomechanical properties similar to those of healthy discs.

In this study, we explore the utilization of mucin hydrogels (Muc‐gels) as a novel approach to protect the immune‐privileged niche post‐discectomy, with the aim of preventing IVD degeneration (**Scheme** **1**). Initially, we assess the presence and phenotypic characteristics of infiltrating macrophages in human IVD samples, distinguishing between healthy/mild (grades I‐III) and severe degenerated (grades IV and V) discs. Subsequently, we evaluate the potential of Muc‐gels, renowned for their immunomodulatory properties based on prior research, to shield explanted discs from immune assaults over 14 days in a mouse subcutaneous model. We unravel the intricate interplay between Muc‐gels and macrophages, aiming to better understand this dynamic interaction. Furthermore, we conduct comprehensive preclinical evaluations utilizing a rat model of IVD degeneration. These evaluations span durations of 4 and 8 weeks and 6 months, enabling us to assess the viability, integrity, and function of the IVD. By undertaking these comprehensive assessments, we aim to shed light on the potential of Muc‐gels in preserving the immune‐privileged niche following discectomy, thereby advancing the field of IVD herniation treatment and offering new avenues for preventing IVD degeneration post‐discectomy.

## 2 Results

### 2.1 Infiltrating Macrophages and their Phenotypes in IVDs

**Figure 1:**
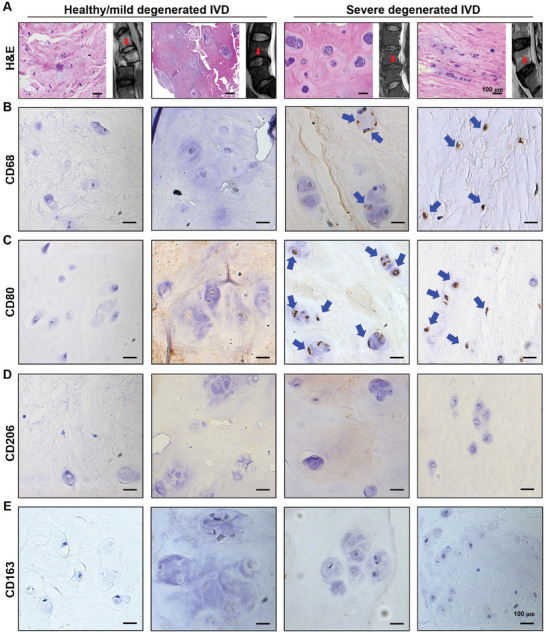
Macrophages are present in human severely degenerated IVDs with only the type I phenotype (M1 macrophages, CD80^+^) but not the type II phenotype (M2 macrophages, CD206^+^ or CD163^+^) as shown by Hematoxylin and Eosin (H&E) staining and immunohistochemical staining. The red arrows in the magnetic resonance imaging (MRI) images indicate the IVD specimens, while the blue arrows in the IHC images indicate positive cells.

We investigate the presence of infiltrating macrophages and their phenotypes in IVDs from patients undergoing IVD surgery, categorizing these IVDs into healthy/mild (grades I‐III) and severe degenerated (grades IV and V) discs (Table S1, Supporting Information). In severe degenerated IVD, macrophages (CD68^+^) are present, significantly exhibiting the type I phenotype (M1 macrophages, CD80^+^) and lacking the type II phenotype (M2 macrophages, CD206^+^ or CD163^+^), as depicted in **Figure** **1** and Figure S1 (Supporting Information). IL‐1β and C‐X‐C motif chemokine ligand 8 (CXCL8) are prominent pro‐inflammatory cytokines implicated in various inflammatory processes. Macrophages are recognized as major producers of IL‐1β and CXCL8.^[^ ^20^, ^29^ ^]^ Upregulation of L‐1β and CXCL8 is reported in degenerated IVDs and can lead to discogenic pain.^[^ ^30^ ^]^ Our findings indicate that levels of IL‐1β and CXCL8 are elevated in severely degenerated IVDs compared to healthy/mildly degenerated IVDs (Figure S2, Supporting Information). Collectively, these results align with the previous research indicating that M1 macrophages contribute to IVD degeneration through their role in fostering an inflammatory environment and subsequent tissue damage.^[^ ^31^ ^]^ Additionally, the absence of M2 macrophages imply a potential deficiency in the regenerative processes and the resolution of inflammation.^[^ ^32^ ^]^ Consequently, we hypothesize macrophages, particularly the M1 phenotype, play a vital role in the pathogenesis of IVD degeneration.

### 2.2 Characterization of Alginate Hydrogels (Alg‐Gels) and Muc‐Gels

**Figure 2:**
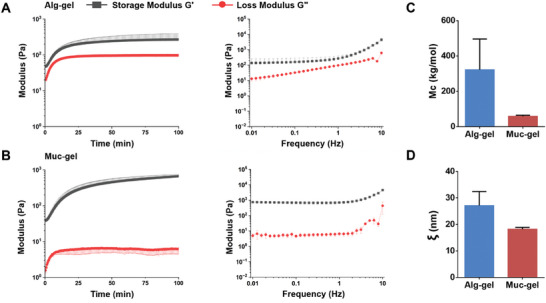
The rheological characterization of Alg‐gels and Muc‐gels is investigated A,B). Time‐dependent rheological assessments (depicted on the left) and frequency‐dependent viscoelastic behaviors (depicted on the right) of Alg‐gels and Muc‐gels were conducted using a rheometer. Standard deviation error bars, derived from measurements performed on three independent samples (n = 3), are included to represent the variability within the dataset. C,D) The estimated average molecular weight between the cross‐links (*Mc*) and mesh size (*ξ*) of Muc‐gels were calculated based on an average of storage modulus (G′) values between 90 to 100 min in the time‐dependent sweep assessment following the Equations (1) and (2). The error bars represent the standard deviation obtained by three independent repeated measurements. Statistical analysis is determined by Prism 9.0 using the unpaired parametric Student's t‐test.

The time‐sweep rheology analysis provides insights into the evolving mechanical properties of both Alg‐gels and Muc‐gels over time. Prior to the onset of measurements, both types of gels have already undergone gelation. As the analysis progressed, a notable and substantial increase in the storage modulus (G') is observed within the following 20 min. Finally, after ≈40 min, the materials reach a state reminiscent of a plateau, signifying that their mechanical properties have achieved stability. A frequency‐sweep analysis is performed after the viscoelastic moduli reaches plateaus, demonstrating that the system indeed appears to be efficiently and covalently crosslinked. This analysis elucidates a more pronounced elastic modulus in Muc‐gels compared to Alg‐gels (**Figure** **2**A,B). The average molecular weight between cross‐links (*Mc*, Equation (1) and Figure 2C) and the average distance between two adjacent cross‐links, mesh size (*ξ*, Equation (2) and Figure 2D) of Alg‐gels are comparable to Muc‐gels. Taken together, these data have revealed that Muc‐gels have similar rheological properties to Alg‐gels. In addition, the results of both in vitro and in vivo degradation tests indicate that Alg‐gels and Muc‐gels are stable (Figure S3, Supporting Information).

### 2.3 Protection IVDs from Macrophage Infiltration and Activation by Muc‐Gels

**Figure 3:**
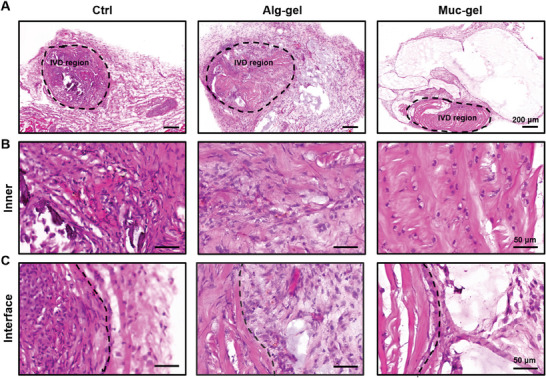
Tissue in‐growth in gel‐encapsulated IVDs, and non‐encapsulated IVDs in a mouse subcutaneous model at week 2 are investigated using HE staining A). Representative images of the internal IVD region B) and the interface between the IVD region and external tissues C).

**Figure 4:**
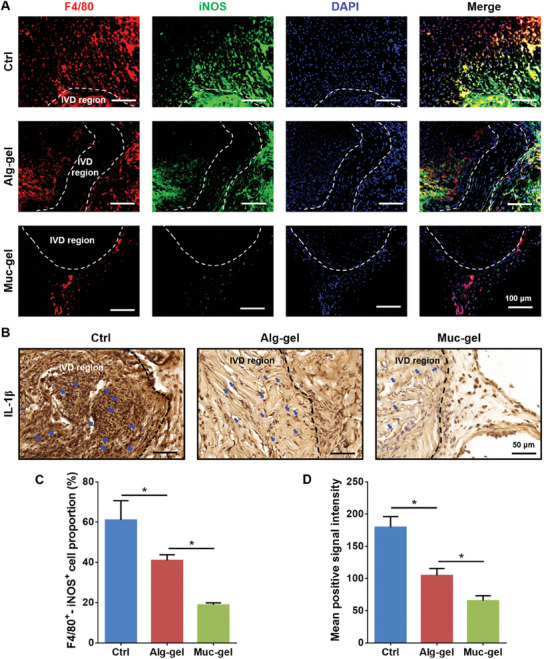
Macrophage infiltration into gel‐encapsulated IVDs, and non‐encapsulated IVDs are observed using immunofluorescent staining in a mouse subcutaneous model at week 2 A), IL‐1β expression is studied with immunohistochemical staining of explants at week 2 B). The M1 proportion of the total cell population C) and the intensity of IL‐1β expression D) are quantified using Image J. The IL‐1β^+^ cells inside the IVD region are indicated by blue arrows. The error bars represent the standard deviation obtained from measurements of n = 9 independent samples from 9 mice, each with three independent repeats. Statistical analysis is performed using ordinary one‐way ANOVA tests with Prism 9.0. *, *p* < 0.05.

Following the observation of macrophage presence in severely degenerated IVDs, especially the M1 phenotype, we hypothesize that infiltration and activation by these cells is a critical factor contributing to IVD degeneration. We proceed to investigate whether Muc‐gels could inhibit M1 macrophage infiltration in gel‐encapsulated IVDs using a mouse subcutaneous model. The findings of our study indicate that Muc‐gels act as distinct barriers, impeding both tissue ingrowth and macrophage infiltration into the gels and encapsulated IVDs by the second week post implantation. This substantiates the preservation of the characteristic low‐cellularity nature of IVDs. In stark contrast, Alg‐gels and non‐encapsulated IVDs (Crtl) show that severe tissue ingrowth and infiltration (**Figure** **3**A–C). We investigate macrophage infiltration into the gels, and find the presence of many M1 macrophages (F4/80^+^ iNOS^+^) in Alg‐gels, and clearly fewer in Muc‐gels (**Figure** **4**A,C; Figure S4A, Supporting Information). Furthermore, the results demonstrate that Muc‐gels inhibit the IL‐1β expression in encapsulated IVDs compared to those encapsulated within Alg‐gels or not encapsulated at all (Figure 4B,D; Figure S4B, Supporting Information). M2‐like macrophages are known for their anti‐inflammatory properties. They can produce anti‐inflammatory cytokines, such as IL‐10, as well as growth factors that contribute to tissue healing and immunosuppression.^[^ ^33^ ^]^ Our results reveal low M2 macrophages (F4/80^+^ CD206^+^) infiltration and anti‐inflammatory cytokine IL‐10 expression across all implants (Figure S4C,D and Figure S5, Supporting Information).

**Figure 5:**
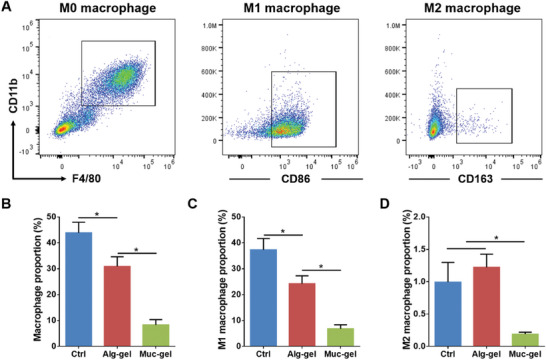
Comparison of macrophage infiltration in gel‐encapsulated IVDs and non‐encapsulated IVDs in a mouse subcutaneous model and their phenotypic characterization by FACS at week 2. A representative FACS profile showing the gating strategy used for the analysis of host macrophage infiltration (F4/80^+^ CD11b^+^) with both M1 (F4/80^+^ CD11b^+^ CD86^+^) and M2 (F4/80^+^ CD11b^+^ CD163^+^) phenotypes A). The proportion of macrophages, including M1 and M2 macrophages, within total cell population retrieved from both gel‐encapsulated and non‐encapsulated IVDs are quantitatively analyzed using FlowJo 10.5.2 B–D). The error bars represent the standard deviation obtained from measurements of n = 9 independent samples from 9 mice, each with three independent repeats. Statistical analysis was performed using ordinary one‐way ANOVA tests with Prism 9.0. *, *p* < 0.05.

Additionally, we perform quantified analysis of the macrophage population and phenotype in Muc‐gel‐ and Alg‐gel‐ encapsulated and non‐encapsulated (Crtl) IVDs at week 2 using flow cytometry (FACS). Our results show Muc‐gels significantly inhibit macrophage infiltration (F4/80^+^ CD11b^+^), including both M1 macrophages (F4/80^+^ CD11b^+^ CD86^+^) and M2 macrophages (F4/80^+^ CD11b^+^ CD163^+^) compared to Alg‐gel and Ctrl groups (**Figure** **5**A–D; Figure S6, Supporting Information). Moreover, in an in vitro experiment, it is found that compared to RAW 264.7 cells seeded on Alg‐gels, those seeded on Muc‐gels exhibit inhibited activation to M1 macrophages when co‐treatment with LPS and IFN‐γ (Figure S7A,B, Supporting Information). Quantitative polymerase chain reaction (qPCR) analysis reveals that the expression of pro‐inflammatory genes (*Il1b*, *Nos2* and *Tnfa*) induced by LPS and IFN‐γ is blocked in the Muc‐gel groups (Figure S7C, Supporting Information). Consistent with qPCR results, Western blot analyses further show that the protein levels of pro‐inflammatory factors (iNOS and Cox‐2) are lower in Muc‐gels compared with Alg‐gels (Figure S7D,E, Supporting Information). These findings provide further evidence that Muc‐gels not only create a physical barrier but also serve as an immune‐suppressive niche which could dampen immune cell recruitment and activation, fostering a more favorable environment for disc degeneration prevention.

### 2.4 Prevention of IVD Degeneration by Muc‐gels in a rat IVD Herniation Model

We then proceed to evaluate the potential efficacy of immediate intervention using Muc‐gels to prevent IVD degeneration in a rat tail IVD degeneration model established through needle puncture. Our comprehensive evaluations covered durations of 4 weeks, 8 weeks, and 6 months, enabling us to assess the long‐term effects of Muc‐gel intervention.

**Figure 6:**
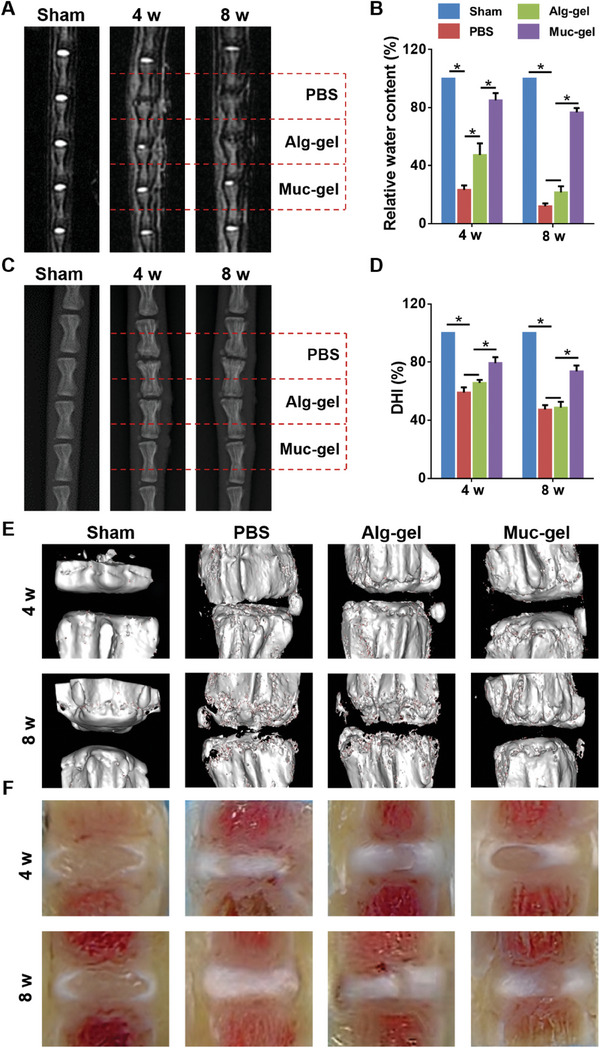
Comparison of immediate interventions with Muc‐gels in a rat tail IVD degeneration model with Alg‐gels as well as PBS and sham groups at weeks 4 and 8. MRI and X‐ray imaging illustrated the relative water content A) and disc height index C) of IVDs respectively, along with their quantification B,D). Micro‐CT scanning and gross morphologies provided morphological images of the vertebrae and IVDs, respectively E,F). The error bars represent the standard deviation obtained from measurements of n = 12 independent samples from 12 rats, each with three independent repeats. Statistical analysis was performed using ordinary one‐way ANOVA tests with Prism 9.0. *, *p* < 0.05.

MRI imaging, a modality renowned for its exceptional sensitivity to water content, is used as the primary approach in this study. Areas of heightened brightness in MRI imaging correlate with water concentration. Muc‐gels show analogous areas of heightened brightness within the NP of IVDs, as evidenced by the increased brightness between vertebrae, which is comparable to the sham group. In stark contrast, groups treated with Alg‐gel or PBS exhibit discernible disparities in this regard (**Figure** **6**A,B). Moreover, Muc‐gels display a comparable disc height index (DHI) of ≈85%, whereas both Alg‐gels and PBS groups demonstrate a significantly reduced DHI when compared to the sham group. (Figure 6C,D). Micro‐CT scanning is a valuable technique that allows for detailed examination and assessment of the microstructure of the vertebrae that are connected by IVDs. The Micro‐CT images reveal Muc‐gels maintain analogous structural integrity of the vertebrae, as compared with the sham group (Figure 6E), suggesting the excellent function of stress dispersion of the disc treatment with Muc‐gels. The gross morphologies of IVDs at 4 and 8 weeks after surgery are shown in Figure 6F. The large and intact NP tissues are seen and an obvious boundary between AF and NP tissues is observed in the Muc‐gel group, while NP loss or atrophy is found in the PBS and Alg‐gel groups. Preserving the integrity of the hard tissue is vital for maintaining the overall structural stability and function of the IVDs. These findings suggest the potential of using Muc‐gels to treat IVD degeneration, which could restore the cushioning function of the IVD toward the vertebra. This restoration may play a preventive role in averting complications such as vertebral bone hyperplasia, endplate fractures, and modic changes.

**Figure 7:**
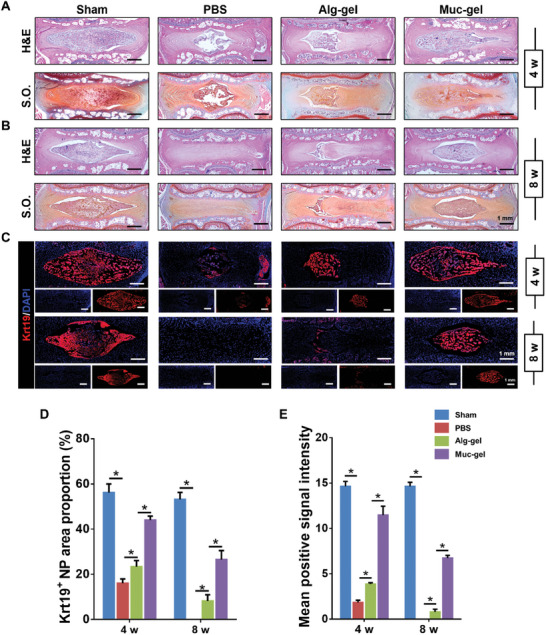
Comparison of immediate interventions with Muc‐gels in a rat tail IVD degeneration model, alongside Alg‐gel, PBS, and sham groups at weeks 4 and 8, using histological and immunofluorescent staining. H&E staining and S.O. staining illustrates histological features of IVDs A,B). Immunofluorescent staining illustrates the expression and spatial distribution of Krt19 C), along with their quantification using Image J D,E). The error bars represent the standard deviation obtained from measurements of n = 12 independent samples from 12 rats, each with three independent repeats. Statistical analysis was performed using ordinary one‐way ANOVA tests with Prism 9.0. *, *p* < 0.05.

Subsequently, the histological features of IVDs are assessed using H&E staining and Safranin O‐fast green (S.O.) staining after 4 and 8 weeks. The H&E staining reveal that the IVDs treated with Muc‐gels exhibit histological features comparable to those of the sham group (**Figure** **7**A). Furthermore, the evaluation using S.O. staining, which specifically highlight proteoglycan content, also demonstrate a resemblance between the Muc‐gel treated group and the sham group (Figure 7B). In contrast, the Alg‐gel and PBS groups show divergent histological characteristics of the IVDs.

Krt19, also known as Keratin 19, is a protein that belongs to the intermediate filament family and is primarily expressed in various epithelial tissues.^[^ ^34^ ^]^ Krt19 serves as a marker for identifying and characterizing NP cells that contribute to the maintenance of the disc's extracellular matrix and its overall health. Remarkably, the NP tissue of PBS group protrude through a needle channel to the outside of the IVD, indicated by Krt19^+^ tissues at the disc periphery at 4 weeks, which is not observed in the Alg‐gel and Muc‐gel groups (Figure 7C). Moreover, the IVDs treated with Muc‐gel exhibit analogous Krt19 expression and spatial distribution, as compared to the sham group. In contrast, the Krt19 expression patterns in Alg‐gel and PBS treatment groups decrease sharply from 4 weeks to 8 weeks (Figure 7C–E). Another potential marker of NP cells, carbonic anhydrase XII (CAXII), has also been investigated.^[^ ^35^ ^]^ Results of CAXII immunofluorescence further indicate that Muc‐gel treatment can maintain the NP phenotype (Figure S8A,B, Supporting Information). These findings highlight that the effective closure of broken AF is the fundamental for preventing NP further loss and delaying the IVD degenerated process, potentially contributing to the physical barrier function of AF to restrict NP protrusion restored by Muc‐gels.

**Figure 8:**
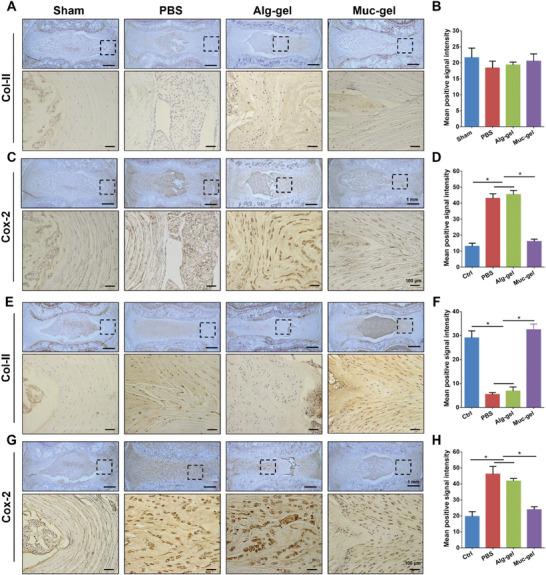
Comparison of Col‐II and Cox‐2 expression levels in immediate interventions with Muc‐gels in a rat tail IVD degeneration model, alongside Alg‐gel, PBS and sham groups. Representative images of the immunohistochemical staining of Col‐II A,E) and Cox‐2 C,G) at 4 and 8 weeks after surgery, respectively, and the semi‐quantitative analysis of Col‐II B,F) and Cox‐2 D,H) at these timepoints. The error bars represent the standard deviation obtained from measurements of n = 12 independent samples from 12 rats, each with three independent repeats. Negative control is shown in Figure S15 (Supporting Information). Statistical analysis was performed using ordinary one‐way ANOVA tests with Prism 9.0. *, *p* < 0.05.

In order to investigate potential mechanisms underlying Muc‐gel‐mediated excellent therapeutic effect, we examine the expression of collagen type 2 (Col‐II), a crucial component of the extracellular matrix in IVDs, and Cox‐2, an enzyme involved in the production of inflammatory prostaglandins, critical parameters of the induction of discogenic pain.^[^ ^36^ ^]^ Our findings show that Muc‐gels effectively reduce inflammation, as evidenced by the reduction in Cox‐2 expression, while simultaneously inhibiting the degradation of the critical extracellular matrix component Col‐II at 8 weeks (**Figure** **8**A–H), both of which have significant implications for the overall preservation and integrity of IVDs.^[^ ^37^ ^]^ Despite sealing the ruptured AF with Alg‐gels, IVDs still exhibit noticeable signs of degeneration. This indicate that Muc‐gels not only seal the ruptured AF but also create an immune‐suppressive niche capable of dampening inflammatory response. These findings underscore the potential of Muc‐gel as a promising intervention strategy in the field of IVD pathology.

**Figure 9:**
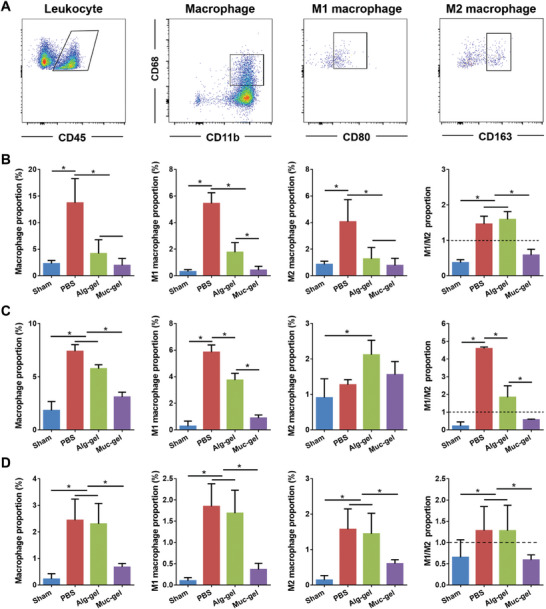
Comparison of macrophage infiltration in immediate interventions with Muc‐gels in a rat tail IVD degeneration model, alongside Alg‐gel, PBS and sham groups and their phenotypic characterization by FACS. A representative FACS profile shows the gating strategy used for the analysis of macrophages infiltration (CD68^+^ CD11b^+^ with both M1 (CD68^+^ CD11b^+^ CD80^+^) and M2 (CD68^+^ CD11b^+^ CD163^+^) macrophages) A). B–D) The proportion of macrophage, including M1 and M2 macrophages within total cell population among all groups are quantitatively analyzed using FlowJo 10.5.2 at weeks 1, 4 and 8, respectively. The error bars represent the standard deviation obtained from measurements of n = 12 independent samples from 12 rats, each with three independent repeats. Statistical analysis was performed using ordinary one‐way ANOVA tests with Prism 9.0. *, *p* < 0.05.

Inflammatory response exists in the pathological process of disc degeneration, closely related to the invasion of immune cells, especially macrophages.^[^ ^20^, ^38^ ^]^ Recent studies have demonstrated that macrophages, in particular, M1 macrophages are the major type of inflammatory cells infiltrated into the degenerated NP tissue.^[^ ^39^ ^]^ We proceed to quantify the macrophage proportion and phenotype in IVD post‐operation using FACS. Our findings exhibit that Muc‐gels significantly inhibit macrophage infiltration (CD68^+^ CD11b^+^), including both M1 macrophage (CD68^+^ CD11b^+^ CD80^+^) and M2 (CD68^+^ CD11b^+^ CD163^+^) macrophages as compared to Alg‐gel and PBS treatments at weeks 1, 4, and 8 (**Figure** **9**A–D; Figures S9–S11, Supporting Information). These quantitative findings provide robust evidence of Muc‐gel's ability to impede macrophage infiltration and modulate their responses.

**Figure 10:**
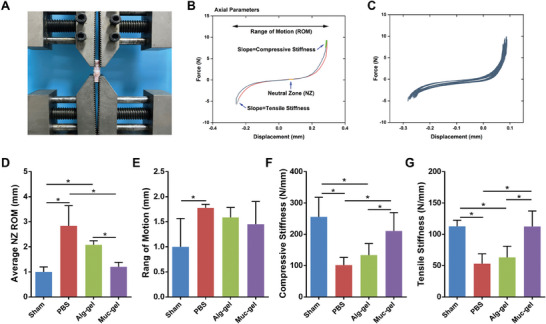
Comparison of the axial range of motion (ROM) and axial neutral zone (NZ) length, coupled with compressive and tensile stiffness of IVDs, following immediate interventions with Muc‐gels in a rat tail IVD degeneration model, alongside Alg‐gel, PBS and sham groups at week 8. A) Axial tension‐compression biomechanical testing. B,C) Representative force‐displacement curve of sham group. Results of torsional neutral zone (NZ) length D), range of motion (ROM) E), compressive stiffness E) and tensile stiffness F). The error bars represent the standard deviation obtained from measurements of n = 20 independent samples from 15 rats, each with five independent repeats. Statistical analysis is performed using ordinary one‐way ANOVA tests with Prism 9.0. *, *p* < 0.05.

The IVD stands as a pivotal structure, contributing significantly to the spine's load‐bearing capacity and flexibility, encompassing both bending and torsional movements.^[^ ^40^ ^]^ IVD degeneration is widely recognized as the cause of their altered biomechanics.^[^ ^41^ ^]^ In light of the potential biomechanical alterations resulting from minor disc injuries and their impact on the microscopic behavior of the IVDs, our study seeks to assess the restoration of biological functions 8 weeks post‐surgery. The evaluation employs composite MN patches in conjunction with biomechanical tests, as illustrated in **Figure** **10**A–C and Figure S12 (Supporting Information). The outcomes of the axial cyclic tension‐compression test disclose a noteworthy increase in both the axial range of motion (ROM) and axial neutral zone (NZ) length within the PBS group. This increase signifies the onset of IVD degeneration when contrasted with the sham group. Notably, the Muc‐gels exhibit a marked reduction in axial NZ length, coupled with minimal decreases in compressive and tensile stiffness (Figure 10D–G). These findings suggest Muc‐gels preserve the biomechanical function, comparable to the characteristics of healthy IVDs. It is imperative to note that a recognized drawback of spinal fusion is the inevitable reduction in spine motion.^[^ ^42^ ^]^ Therefore, the application of Muc‐gel on disc herniation undergoing surgical procedures presents a potential avenue for the preservation of biomechanical function and enhancement of spine mobility.

In addition, our study provides long‐term evidence that the protective effects of Muc‐gels could be sustained for a duration of up to 6 months, as supported by higher water content and disc height (Figure S13, Supporting Information). From a microscopic perspective, the results of histological evaluation finds that the NP tissue disappeared completely in the PBS and Alg‐gel groups, while IVDs treated with Muc‐gels preserve partial NP tissue, represented by highly positive Krt19 and CAXII expression levels (Figures S14A–D and S8C, Supporting Information). Furthermore, the expression of Col‐II and Cox‐2 is comparable to the levels observed in the sham group. Inversely, the lower Col‐II expression and higher Cox‐2 level are seen within the IVDs with PBS and Alg‐gel treatments (Figure S14E–H, Supporting Information). These findings reveal the IVDs in better health maintained by Muc‐gels with balanced matrix metabolism and inflammatory response. This may be attributed to the fact that Muc‐gels re‐build the homeostasis of immune privilege of the disc, both in mechanical and immunological aspects. However, it is crucial to acknowledge that the effectiveness of the treatment may diminish as the Muc‐gel degrades over time. Consequently, it is advisable for individuals to be aware of the possibility of necessitating multiple injections or additional treatments to sustain the benefits in delaying IVD degeneration following discectomy. By considering such measures, the long‐term efficacy of the treatment could be better ensured.

## 3 Discussion

This study investigates the potential of in situ gelling Muc‐gels at surgical sites of IVDs, as immediate intervention in a rat caudal IVD degeneration model. Our findings show that Muc‐gels can prevent the acute IVD degeneration, by diminishing the infiltration and activation of M1 macrophages and impeding the inflammatory cascade. Microdiscectomy for herniated IVD is one of the most frequently performed surgical procedures and is effective in relieving pain.^[^ ^43^ ^]^ When herniated IVDs are removed, the residuals of NP are exposed to the immune system, leading to infiltration of immune cells like macrophages and lymphocytes through broken AF, damaging material. This triggers an immune response, including the release of enzymes and reactive oxygen species, ultimately causing NP deterioration, disruption of nutrient supply, and worsening degeneration and segmental deterioration. Therefore, a significant number of patients experience chronic pain and recurrent IVD herniation, affecting up to 25% of cases,^[^ ^23b^ ^]^ or the progressive reduction of disc height by more than 25%.^[^ ^44^ ^]^ The emergence of tissue engineering holds promises for AF regeneration and repair. However, challenges such as the security of stem cells,^[^ ^45^ ^]^ the limited regenerative capacity of the IVDs, and the inhospitable regenerative milieu significantly impede its development and clinical translation.^[^ ^46^ ^]^ Consequently, there is a large unmet clinical need for interventions that can protect IVD from degeneration following discectomy.

Our findings demonstrate M1 macrophages infiltrate in severely degenerated human IVDs, aligning with a previous study by Ling et al.,^[^ ^47^ ^]^ which utilized single cell sequencing revealed the positive correlation of M1 macrophages in severity of human IVD degeneration. Furthermore, accumulative investigations have reported M1 macrophages are involved in IVD degeneration in various animal models, including mouse,^[^ ^48^ ^]^ rat^[^ ^49^ ^]^ and dog^[^ ^50^ ^]^ models. The inflammatory cytokines (IL‐1β and CXCL8) secreted by M1 macrophages have been found in high levels in severely degenerated human IVDs. This underscores M1 macrophages are significant contributors to IVD degeneration. Our findings also show Muc‐gels significantly inhibit M1 macrophage infiltration and activation, in stark contrast to non‐encapsulated and Alg‐gel‐encapsulated IVDs in a mouse subcutaneous model, which extend in a rat tail IVD degeneration model. Our results demonstrate that Muc‐gels serve not only as a physical shielding barrier but also as an immune‐isolating barrier for NP, as Alg‐gels with similar rheological properties and stability failed to protect against IVD degeneration. Muc‐gels effectively suppress proinflammatory cytokine secretion such as IL‐1β and Cox‐2, which are primarily produced by activated M1 macrophages, initiating inflammatory cascades,^[^ ^51^ ^]^ thereby perpetuating a deleterious cycle that accelerates IVD degeneration. Cox‐2 inhibitor such as ibuprofen has been used to control the inflammation to diminish IVD degeneration. The results further support our earlier investigations, demonstrating that Muc‐gels suppress immune cell infiltration and activation at the implantation site, as well as immune cell activation under various circumstances,^[^ ^12^ ^]^ which may be attributed to Muc‐gels having a rich array of glycan structures that can bind to surface receptors, such as galectins and Siglecs, thereby exerting immunomodulatory functions.

Additionally, our findings demonstrate that M2 macrophages rarely infiltrate human IVDs, as well as specimens from both mice and rat models. This observation is consistent with the limited regenerative capacity of IVDs, where attempts to promote regeneration are made with limited success. Mostly, studies show M2 macrophages mitigate inflammation in degenerated IVDs and improve IVD health.^[^ ^52^, ^53^ ^]^

Integral to the spinal function is its capacity to adeptly respond to diverse and intricate mechanical loads. The mechanical resilience exhibited by IVDs assumes a central role in ameliorating the imposed load on the spine, consequently exerting a discernible impact on the integrity of the contiguous vertebral interfaces. Following discectomy, the application of Alg‐gel intervention has demonstrated a partial mitigation of degenerative changes. Notwithstanding these positive effects, it is crucial to note that Alg‐gel fails to preserve the native IVD mechanical properties. The dehydrated NP tissue, damaged AF, and degradative matrix contribute to a compromise in the proper mechanical function of IVDs. In contrast, the introduction of Muc‐gels post‐discectomy facilitates the restoration of hydration and matrix synthesis within the NP. Consequently, 6 months post‐application, Muc‐gel injection proves instrumental in maintaining the native morphology and functional mechanical properties of IVDs in vivo. This robustly underscores the pivotal role of Muc‐gels in filling lesions in the AF and retarding the disc degeneration, presenting a promising avenue for application in microdiscectomy. Recently, several AF repair technologies have been proposed and applied to prevent re‐herniation, including the Barricaid annular closure device and AF suture system (Xclose system and annular stapler).^[^ ^54^ ^]^ The application of the Barricaid system in lumbar discectomy has demonstrated efficacy in reducing postoperative recurrence rates. However, it is crucial to acknowledge potential risks associated with this intervention, including the possibility of device loosening, displacement, breakage, and damage to surrounding tissues.^[^ ^55^ ^]^ Although the safety of physical suture methods in clinical practice has been validated, the clinical outcomes of these techniques remain suboptimal.^[^ ^56^ ^]^ Furthermore, sealants have been emerging in this domain, such as fibrin sealants, aimed at sealing AF lesions. Despite the convenience of direct injection into the injured site offered by sealants, they exhibit shortcomings in addressing the IVD mechanical function.^[^ ^57^ ^]^ All current AF repair technologies still have deficiencies, biomaterials with better performance will be the main direction of future development.

Muc‐gels show the promising potential of for preclinical and clinical development, yet this study is not devoid of limitations. Given the inherent differences between rats and humans, the inability to fully replicate the mechanical environment following microdiscectomy necessitates the optimization of animal models to enhance clinical translation. Although our in vivo pilot is limited in model size, signs of therapeutic benefits are apparent up to 6 months. In the future, a large animal model will determine whether these signs of improved degeneration result following microdiscectomy. In addition, compressive loading and tensile loading are investigated in ex vivo mechanical testing, while torsional stiffness and torque range aren't tested, which is highly sensitive to discectomy injuries. Future work will need to assess the effects of multiaxial loading on the maintenance of IVD mechanical properties by Muc‐gel intervention. Taken together, this study elucidates the dual roles of Muc‐gels in physical and immune blocking, offering a novel approach to diminish the recurrence rate of discectomy and delay disc degeneration.

## 4 Conclusion

In this study, a robust physical and immune barrier is realized through the utilization of Muc‐gels. These gels are fabricated by employing click chemistry to conjugate Tz‐modified mucin and Nb‐modified mucin, achieving gelation within 3 to 5 min. This property allows Muc‐gels solidify in situ upon injection into the surgical site within damaged AF tissue. Notably, our findings unequivocally demonstrate that the administration of Muc‐gels at the surgical locus effectively averts NP herniation and IVD degeneration. This is substantiated by the sustained maintenance of normal relative water content within the NP, preservation of disc height index, structural integrity, and the observation of biomechanical properties akin to those exhibited by healthy intervertebral discs.

## 5 Experimental Section

### Materials

Amine derivatives of tetrazine (Tz) and norbornene (Nb) were acquired from the Bioconjugate Technology Company and TCI Europe N.V., respectively. All other chemicals were purchased from Sigma–Aldrich, a reputable supplier of laboratory chemicals and reagents.

### Hydrogel Preparation

Hydrogels derived from mucin (referred to as Muc‐gels) were synthesized in accordance with previously documented procedures. To provide a succinct overview, bovine submaxillary mucin (BSM) underwent predissolution in MES buffer (0.1 M MES, 0.3 M NaCl, pH 6.5) at a concentration of 10 mg mL^−1^. Subsequently, 1‐ethyl‐3‐(3‐dimethylaminopropyl) carbodiimide (EDC) and N‐hydroxysuccinimide (NHS), each quantified at 4 mmol per gram of dry mucin, were introduced into the mixture and agitated for a duration of 15 min. Following this, 1 mmol of tetrazine‐amine (Tz) and 2 mmol of norbornene‐amine (Nb) were individually incorporated to produce BSM tetrazine (BSM‐Tz) or BSM norbornene (BSM‐Nb). The resulting blends underwent an overnight stirring process at 4 °C, followed by a subsequent 4‐day dialysis. The resultant samples underwent freeze‐drying and were subsequently preserved at −20 °C. The components facilitating gelation were each pre‐dissolved in a phosphate‐buffered saline (PBS) solution at a concentration of 45 mg mL^−1^. Alginate‐based hydrogels (referred to as Alg‐gels) were prepared using methodologies akin to those elucidated above.

### Rheological Characterization of Hydrogels

Evaluation of the Alg‐gel and Muc‐gel rheological proprieties was performed using a rheometer (Kinexus Prot, Malvern). 8 mm diameter parallel plates and 1 mm gap size was applied for all the measurements. Before the measurements, Muc‐Tz and Muc‐Nb as well as Alg‐Tz and Alg‐Nb underwent predissolution in a PBS solution with a pH of 7.4, maintaining a concentration of 45 mg mL^−1^. The two constituents (Tz and Nb counterparts) underwent thorough mixing, and subsequently, 60 µL of the resultant sample was dispensed onto the rheometer plate. Gelation kinetic analysis and determination of both the storage modulus (G') and loss modulus (G″) was performed immediately after mixing by time sweep spanning t = 100 min – setting the strain to 1% and frequency to 1 Hz. Subsequent to this, an evaluation of frequency‐dependent viscoelasticity occurred via a strain‐controlled (1%) frequency sweep, ranging from f_start_ = 10 Hz to fend = 0.01 Hz. All assays were performed at room temperature with three independent experiments and using mineral oil (Fisher Scientific) as solvent trap in the bottom plate. Both the average molecular weight (*Mc*, the molecular weight of chain segments between two adjacent cross‐links or entanglement points) and mesh size (*ξ*, the distance between two adjacent cross‐links or entanglement points) are calculated following below equations.

(1) $$Mc=cpRT/G_{P}^{\prime }$$

(2) $$\xi ={\left(G^{\prime }PNA/RT\right)}^{-1/3}$$

where *c* is the concentration of polymers (2% w/v), *ρ* is the density of water at 298 K (997 kg m^−3^), *R* is the molar gas constant (8.314 × 10^6^ cm^3^ Pa K mol^−1^), *T* is the temperature (298 K), *G′_P_* is the plateau value of elastic modulus, and *N_A_* was the Avogadro constant.

### Degradation Test of Hydrogels

The initial dry weights of the Alg‐gels and Muc‐gels, denoted as *W_0_*, were measured. Subsequently, 100 µL of each hydrogel was immersed in 5 mL of PBS and placed on a shaker at 37 °C, operating at 60 rpm min^−1^. At predetermined time intervals (1, 2, and 3 w, and 4 s), the hydrogels were removed from the PBS, freeze‐dried, and weighed, with the resulting weights recorded as *W*_t_. The degradation rate was then calculated using the following equation.

(3) $$\mathrm{Degradation}\;\left(\%\right)=\left(W_{t}-W_{0}\right)/W_{0}\times 100\%$$

In addition, the in vivo degradation behavior of the hydrogels was further implanted into the dorsal side of Sprague‐Dawley (SD) rats. The rats were sacrificed at 1, 2, and 4 w, and the implanted site and surrounding tissues were isolated and observed.

### Human Specimens

The study was conducted with the approval of the Ethics Committee of First Affiliated hospital of Soochow University (No. 2023334). Individuals who intended to undergo IVD surgery and were willing to take part in this study signed a written informed consent. The basic characteristics and information for the participants was listed in Table S1 (Supporting Information). In the study, a 5‐level Pfirrmann grading system was applied to evaluate IVD degeneration. Pfirrmann grade I was considered healthy IVD, Pfirrmann grades II and III were defined as mild degeneration, and Pfirrmann grades IV and V were defined as severe degeneration. Immunohistochemical staining were carried out to investigate macrophage infiltration and inflammatory response.

### Macrophage Differentiation In Vitro

RAW 264.7 cells (5 × 10^3^ cells per well) were seeded and cultured on Alg‐gels and Muc‐gels in a 24‐well plate. After adhesion, LPS (10 ng mL^−1^, Sigma–Aldrich, St. Louis, MO, USA) and IFN‐γ (50 ng mL^−1^, R&D Systems, Minneapolis, MN, USA) were used to induce RAW 264.7 cells to differentiate into M1 macrophages. After 12 h treatment, cells were collected for follow‐up experiments. M1 phenotypic characterization was investigated by FACS and the expression levels of M1 macrophage‐associated inflammatory genes and proteins were tested using qPCR and Western blot analyses, respectively. The primer sequences of the genes (Sangon Biotech, Shanghai, China) are listed in Table S2 (Supporting Information), each gene expression was normalized by GAPDH and calculated using the 2^−ΔΔCT^ method. The antibodies of iNOS and Cox‐2 were purchased from Abcam Company.

### Animal Experiment

All animal‐related procedures followed the NIH Guide for the Care and Use of Laboratory Animals and were approved by the Institutional Animal Care and Use Committee of Soochow University (SUDA 20221229A08). For subcutaneous implantation, C57BL/6 mice (8 weeks old, male) were sacrificed for the IVD tissue isolation. After encapsulation with Alg‐gel or Muc‐gel (50 µL per IVD), the allogeneic IVD tissues were implanted subcutaneously into C57BL/6 mice. Subcutaneous implants were taken out after 2 weeks for subsequent analysis. For IVD degenerated models, coccygeal vertebral degeneration models of rats were established using a previously described method. Male SD rats aged 8–10 weeks and weighing 250–300 g were anesthetized with pentobarbital and disinfected by iodophor. A longitudinal incision was made at the midline of the rear side of the tail to expose the discs in the segments of coccygeal vertebrae 7–8 (Co7‐8), 8–9 (Co8‐9), and 9–10 (Co9‐10). These discs were punctured into the middle of the NP tissue with a 21 G needle at a depth of 5 mm. The PBS, Alg‐gel, and Muc‐gel with a volume of 20 µL were injected into the discs of Co7‐8, Co8‐9, and Co9‐10, respectively, along the needle path using 33 G needles. After the suture, rats were transferred to an airy and warm environment to wake up. At 4, 8, and 24 weeks after surgery, disc samples were collected for subsequent analysis.

### Flow Cytometry

To obtain the subcutaneously implanted IVD tissues from mice, the animals were euthanized, and samples were meticulously dissected from the implantation site to remove surrounding tissues. For the collection of rat IVD samples, the rats were euthanized, and the tail skin was removed. The fascia and muscles were then separated to fully expose the IVD tissues. The vertebral body was excised along the junction of the vertebral body and the IVD in a direction parallel to the cartilage endplate, using a blade to obtain complete IVD tissues. The obtained samples were minced and digested with 2 mg mL^−1^ collagenase I and collagenase II (Yeasen, Shanghai, China) at 37 °C for 4 h. The cell suspension was filtered through 100 µm cell strainers to achieve a single‐cell suspension. Erythrocytes were removed by red blood cell lysis buffer (Beyotime, Shanghai, China). After being blocked, cells were stained with an antibody cocktail in the dark at 4 °C for 30 min. The antibody cocktail contained the following fluorescent conjugated antibodies for mouse samples: F4/80‐PE (1 µL per 1 million cells in 100 µL staining volume, Biolegend, San Diego, CA, USA), CD11b‐Alexa Fluor 700 (1 µL per 1 million cells in 100 µL staining volume, Biolegend, San Diego, CA, USA), CD86‐APC/Cy7 (1 µL per 1 million cells in 100 µL staining volume, Biolegend, San Diego, CA, USA), CD163‐APC (1 µL per 1 million cells in 100 µL staining volume, Biolegend, San Diego, CA, USA). The antibody cocktail contained the following fluorescent conjugated antibodies for rat samples: CD45‐Alexa Fluor 700 (1 µL per 1 million cells in 100 µL staining volume, Biolegend, San Diego, CA, USA), CD68‐APC (1.25 µL per 1 million cells in 100 µL staining volume, NOVUS, Littleton, CO, USA), CD11b/c‐PE/Cy7 (1.25 µL per million cells in 100 µL staining volume, Biolegend, San Diego, CA, USA), CD80‐PE (1 µL per million cells in 100 µl staining volume, Biolegend, San Diego, CA, USA), CD163‐APC/Cy7 (1.25 µL per 1 million cells in 100 µL staining volume, NOVUS, Littleton, CO, USA). Cells were washed with 1 mL PBS twice and to be sorted using with a flow cytometer (Thermo Fisher Scientific, Waltham, MA, USA). Data were analyzed with Flowjo 10.6.2 Software (Tree Star, Ashland, OR, USA).

### Imaging Analysis

Magnetic resonance imaging (MRI) scanning of the caudal spines was taken to evaluate the water content of the NP tissues according to the signal intensity in sagittal T2‐weighted images using a 3.0 T clinical magnet (Siemens, Berlin, German). The water content was quantified using ImageJ.

X‐ray imaging of the caudal spines was performed to calculate the disc height index (DHI, Figure S16, Supporting Information), as previously reported.^[^ ^58^ ^]^ The DHI (%) was defined as the ratio of the DHI in the surgical groups to the DHI in the sham group.

A micro‐CT scanner (65 kV, 385 mA, and 1 mm Al filter, SkyScan 1176, SkyScan, Aartselaar, Belgium) was used to evaluate the 3D structure of the adjacent vertebrae of the experimental IVDs.

### Histological Evaluation

The collected mouse specimens underwent fixation in 10% formalin for a duration of 24 h, followed by embedding in optimum cutting temperature compound (OCT, SAKURA, Japan) and horizontal sectioning into 10 µm thick slices. Subsequently, the samples underwent PBS washing for hematoxylin and eosin (H&E) staining. Dehydration and coverslip mounting ensued for the prepared slides. Utilizing a Zeiss microscope equipped with 2.5X and 40X objective lenses (Zeiss Axiovert 200, Carl Zeiss Inc., Thornwood, NY, USA), images were captured. In the case of immunofluorescence, mouse samples underwent PBS washing, a 5% goat serum blocking step, and subsequent staining with F4/80 (1:400, Abcam, Cambridge, UK), iNOS (1:500, Abcam, Cambridge, UK), and CD206 (1:200, Santa Cruz, CA, USA) antibodies to explore macrophage polarization. Fluorescent labeling of F4/80, iNOS, and CD206 was achieved using Cy3‐labeled goat anti‐rat secondary antibody, FITC‐labeled goat anti‐rabbit secondary antibody, and FITC‐labeled goat anti‐mouse secondary antibody (1:800, Beyotime, Shanghai, China), respectively. Nuclei were counterstained with DAPI, and images were acquired employing a fluorescence microscope. Quantitative analysis was conducted using Image J software (NIH, Bethesda, MD, USA). Furthermore, the specimens underwent staining for IL‐1β and IL‐10 for immunohistochemical analysis, with results subjected to semiquantitative evaluation using Image J.

Rat specimens underwent fixation in 10% formalin for 24 h, followed by a one‐month decalcification at 4 °C in a 10% EDTA solution. Subsequently, PBS washing, and gradient alcohol dehydration preceded paraffin embedding. Horizontal cutting into 5‐µm thick slices was performed on the specimens, which were then deparaffinized and rehydrated for H&E and Safranin O‐Fast green (S.O.) staining. In the case of immunofluorescent staining, antigen retrieval was achieved through treatment with 0.025% trypsin solution at 37 °C for 15 min following deparaffinization and rehydration. Blocking nonspecific protein binding was carried out using 5% goat serum diluted in PBS for 1 h at room temperature, and Krt19 antibody (1:200, Abclonal, Boston, MA, USA) and CAXII antibody (1:100, Proteintech, Chicago, IL, USA) were incubated overnight at 4 °C. Subsequent steps involved treatment with cy3 or FITC‐labeled goat anti‐rabbit secondary antibody (1:800, Beyotime, Shanghai, China), and nuclei were counterstained with DAPI. Image acquisition utilized an immunofluorescent microscope (Zeiss Axiovert 200, Carl Zeiss Inc., Thornwood, NY, USA). For immunohistochemistry, sections underwent deparaffinization and rehydration, followed by the antigen retrieval procedure. Blocking endogenous peroxidase utilized 3% (v/v) hydrogen peroxide at room temperature for 20 min. After blocking nonspecific protein binding, samples were incubated with Col‐II (1:200, Abcam, Cambridge, UK) and Cox‐2 (1:400, Abcam, Cambridge, UK) antibodies at 4 °C overnight. Horseradish peroxidase‐conjugated secondary antibodies (1:50, Beyotime, Shanghai, China) were applied to combine with primary antibodies at room temperature for 1 h. Visualization with diaminobenzidine‐based peroxidase substrate (DAB) and counterstaining with hematoxylin were followed by image acquisition using a Zeiss microscope. Quantitative analysis of immunofluorescence and immunohistochemistry was conducted using Image J.

### Biomechanical Tests

Biomechanical attributes of the IVDs, encompassing compressive stiffness, tensile stiffness, range of motion (ROM), and axial neutral zone (NZ) length, were ascertained through axial tension–compression assessments employing a universal material testing machine (Shanghai Hengyi, China). The axial stiffness and NZ length were derived from the concluding full test cycle. Compressive and tensile stiffness were computed as the gradient of the upper 20% segment within the linear phase of the power‐displacement curve. ROM represented the overall displacement from maximum tension to maximum compression. NZ was determined using the stiffness threshold methodology as delineated. The axial NZ stiffness and NZ length were defined by the slope and axial displacement of the NZ region, respectively.

### Statistical Analysis

All experiments were repeated at least three times. All data are provided as the mean ± standard deviation. Statistical analyses were performed with unpaired parametric Student's t‐test for two‐group comparisons and one‐way ANOVA, followed by Tukey post hoc comparison, for multigroup comparisons using GraphPad Prism 9 software (GraphPad Software). A difference was considered statistically significant if p was less than 0.05.

## Conflict of Interest

The authors declare no conflict of interest.

## Supporting information

Supporting Information

## Data Availability

The data that support the findings of this study are available from the corresponding author upon reasonable request.
