# Supplementary material for: Preserving the Immune‐Privileged Niche of the Nucleus Pulposus: Safeguarding Intervertebral Discs from Degeneration after Discectomy with Synthetic Mucin Hydrogel Injection
Source: Adv Sci (Weinh). 2024 Aug 29;11(43):2404496. doi: 10.1002/advs.202404496 (PMC11578300; doi:10.1002/advs.202404496)
Supplement: Supplementary file 1 — Supporting Information [file ADVS-11-2404496-s001.docx]

Supplementary Materials

**Table S1**

**Table S1.** Basic information and characteristics for participants.

| **Patient ID** | **Age (y)** | **Gender** | **Reason for surgery** | **Pfirrmann grading** |
| --- | --- | --- | --- | --- |
| P1 | 18 | male | Cervical vertebral fracture | I |
| P2 | 42 | female | Lumbar disc herniation | II |
| P3 | 34 | male | Lumbar disc herniation | II |
| P4 | 64 | female | Lumbar disc herniation | III |
| P5 | 51 | male | Lumbar disc herniation | III |
| P6 | 47 | male | Lumbar disc herniation | IV |
| P7 | 43 | male | Lumbar disc herniation | IV |
| P8 | 48 | male | Lumbar disc herniation | IV |
| P9 | 59 | female | Lumbar disc herniation | V |
| P10 | 74 | female | Lumbar disc herniation | V |

**Table S2**

**Table S2**. Primers for qPCR.

| **Gene** | **Forward（5’-3’）** | **Reverse（5’-3’）** |
| --- | --- | --- |
| *Tnfa* | CCACCACGCTCTTCTGTCTACTG | TGGTTTGTGAGTGTGAGGGTCTG |
| *Il1b* | CTCGCAGCAGCACATCAACAAG | CCACGGGAAAGACACAGGTAGC |
| *Nos2* | GACGAGACGGATAGGCAGAGATTG | AACTCTTCAAGCACCTCCAGGAAC |
| *Gapdh* | GGTTGTCTCCTGCGACTTCA | TGGTCCAGGGTTTCTTACTCC |

**Fig. S1**


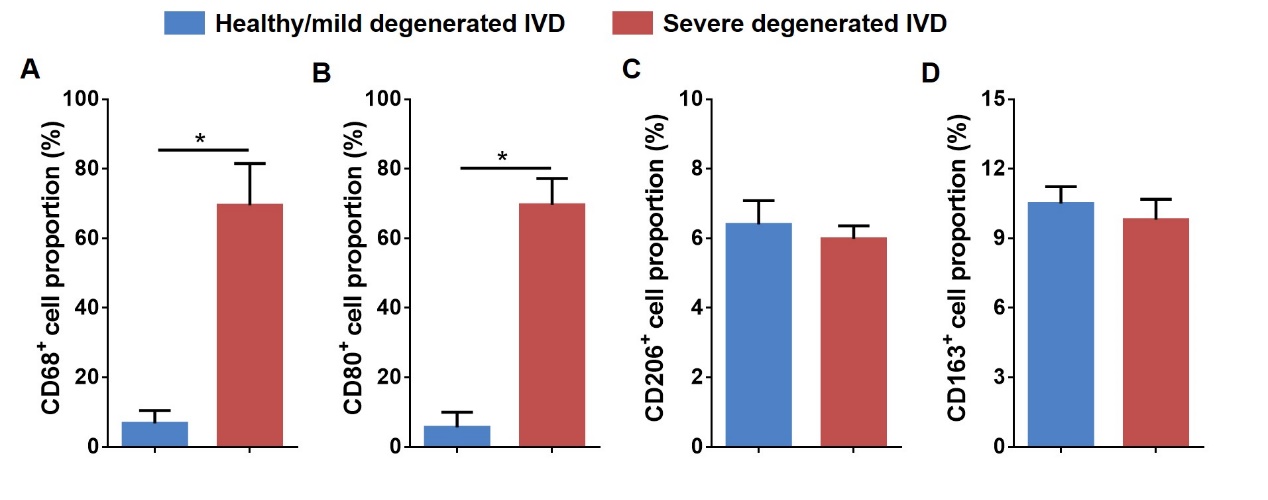


**Fig. S1.** Semi-quantitative analysis for immunohistochemical staining for CD68 (A), CD80 (B), CD206 (C), and CD163 (D), respectively. The error bars represent the standard deviation obtained from measurements of n = 10 independent samples from 10 patients, each with three independent repeats. Statistical analysis was determined by Prism 9.0 using the unpaired parametric Student’s t-test. *, *p* < 0.05.

**Fig. S2**

**
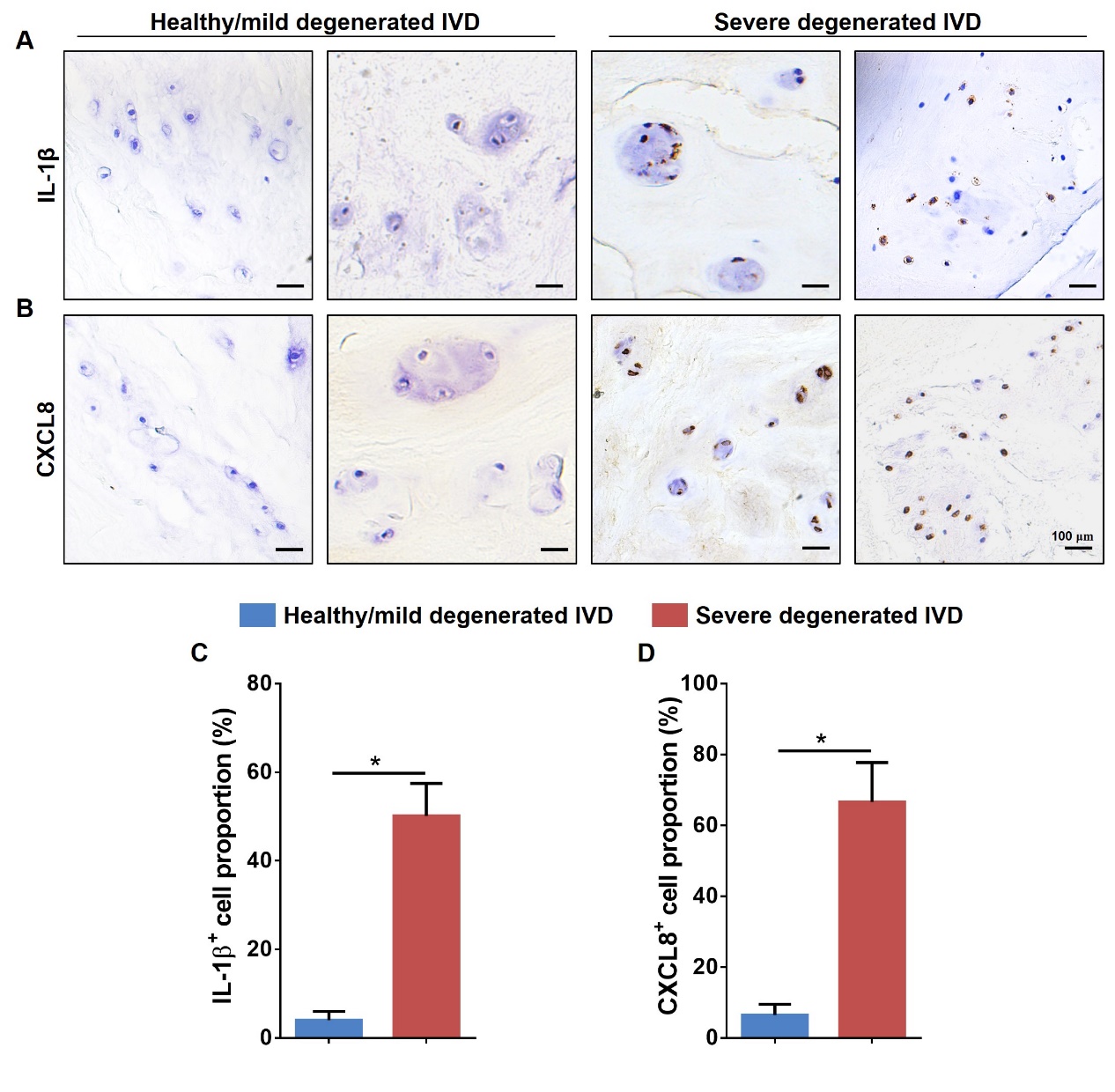
**

**Fig. S2.** Comparison of IL-1β and CXCL8 expression levels in human degenerated IVDs. Representative images of the immunohistochemical staining of IL-1β (A) and CXCL8 (B), respectively, and the semi-quantitative analysis of IL-1β (C) and CXCL8 (D). The error bars represent the standard deviation obtained from measurements of n = 10 independent samples from 10 patients, each with three independent repeats. Statistical analysis was determined by Prism 9.0 using the unpaired parametric Student’s t-test. *, *p* < 0.05.

**Fig. S3**


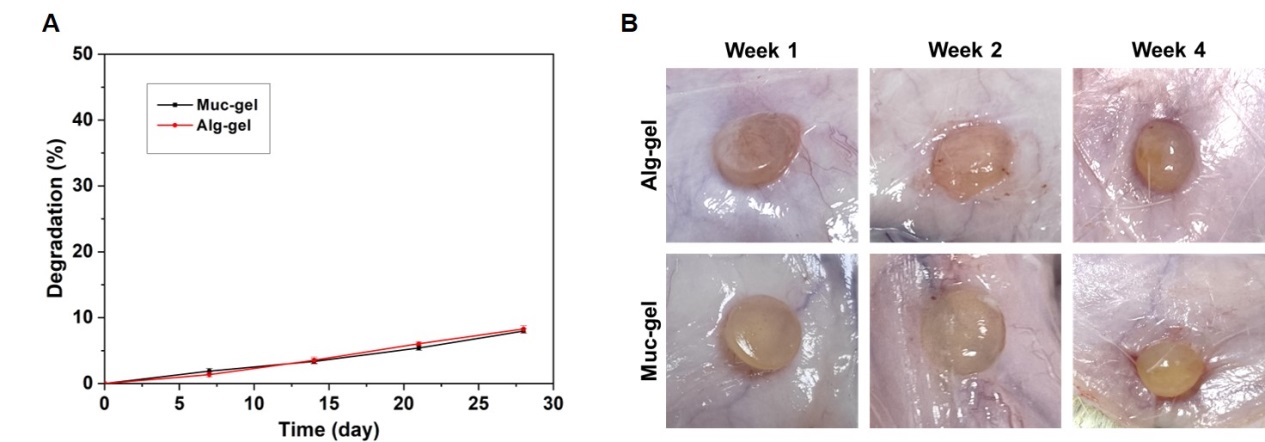


**Fig. S3.** Degradation test of Alg-gels and Muc-gels. (A) Degradation profiles of Alg-gels and Muc-gels upon incubation in PBS at 37 °C for weeks 1, 2, 3 and 4, (n = 3); (B) Gross observation of *in vivo* degradation of Alg-gels and Muc-gels after subcutaneous implantation in rats at weeks 1, 2 and 4.

**Fig. S4**

**
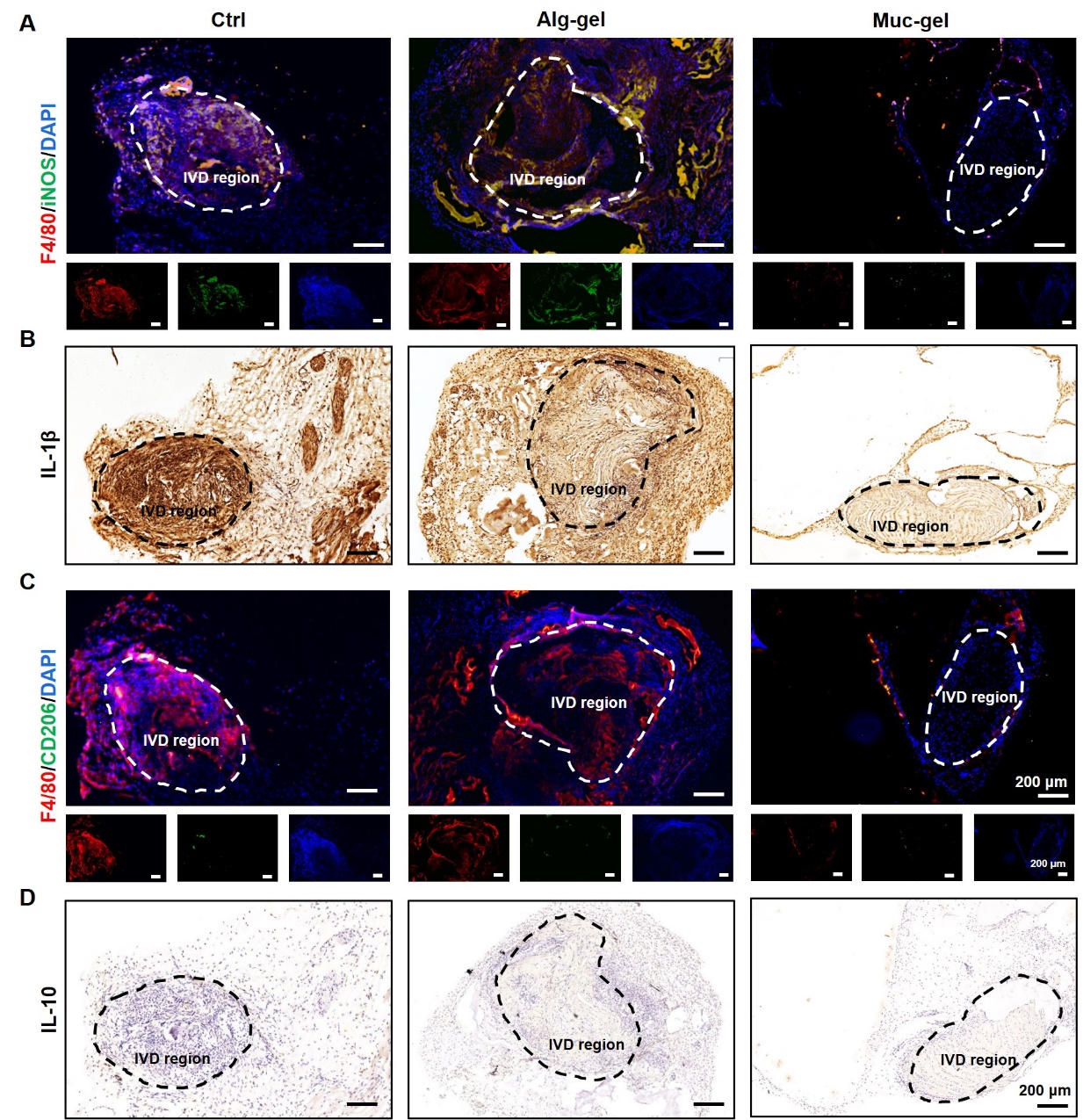
**

**Fig. S4.** Macrophage infiltration into gel-encapsulated IVDs and non-encapsulated IVDs in a mouse subcutaneous model are studied at week 2. (A) The complete tissue field of immunofluorescent staining of explants for observing M1 macrophage infiltration. (B) The complete tissue field of immunohistochemical staining of explants for observing IL-1β expression. (C) The complete tissue field of immunofluorescent staining of explants for observing M2 macrophage infiltration. (D) The complete tissue field of immunohistochemical staining of explants for observing IL-10 expression.

**Fig. S5**


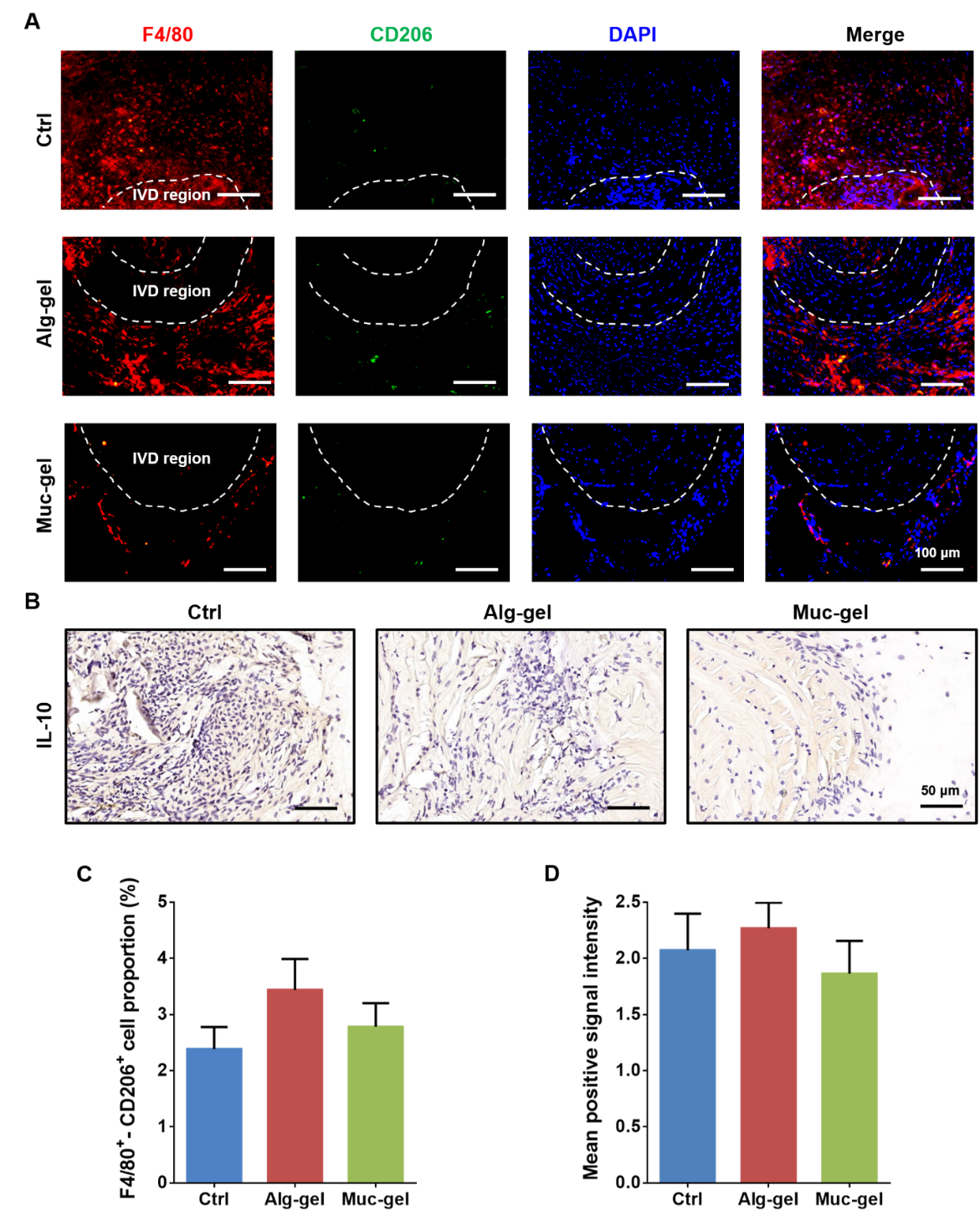


**Fig. S5.** M2 macrophage infiltration into gel-encapsulated IVDs, and non-encapsulated IVDs in a mouse subcutaneous model are studied using immunofluorescent staining of explants at week 2 (A). The IL-10 expression is studied using immunohistochemical staining of explants at week 2 (B). The M2 proportion of the total cell population (C) and the intensity of IL-10 expression (D) are quantified using Image J. The error bars represent the standard deviation obtained from measurements of n = 12 independent samples from 12 mice, each with three independent repeats. Statistical analysis was performed using ordinary one-way ANOVA tests with Prism 9.0.

**Fig. S6**


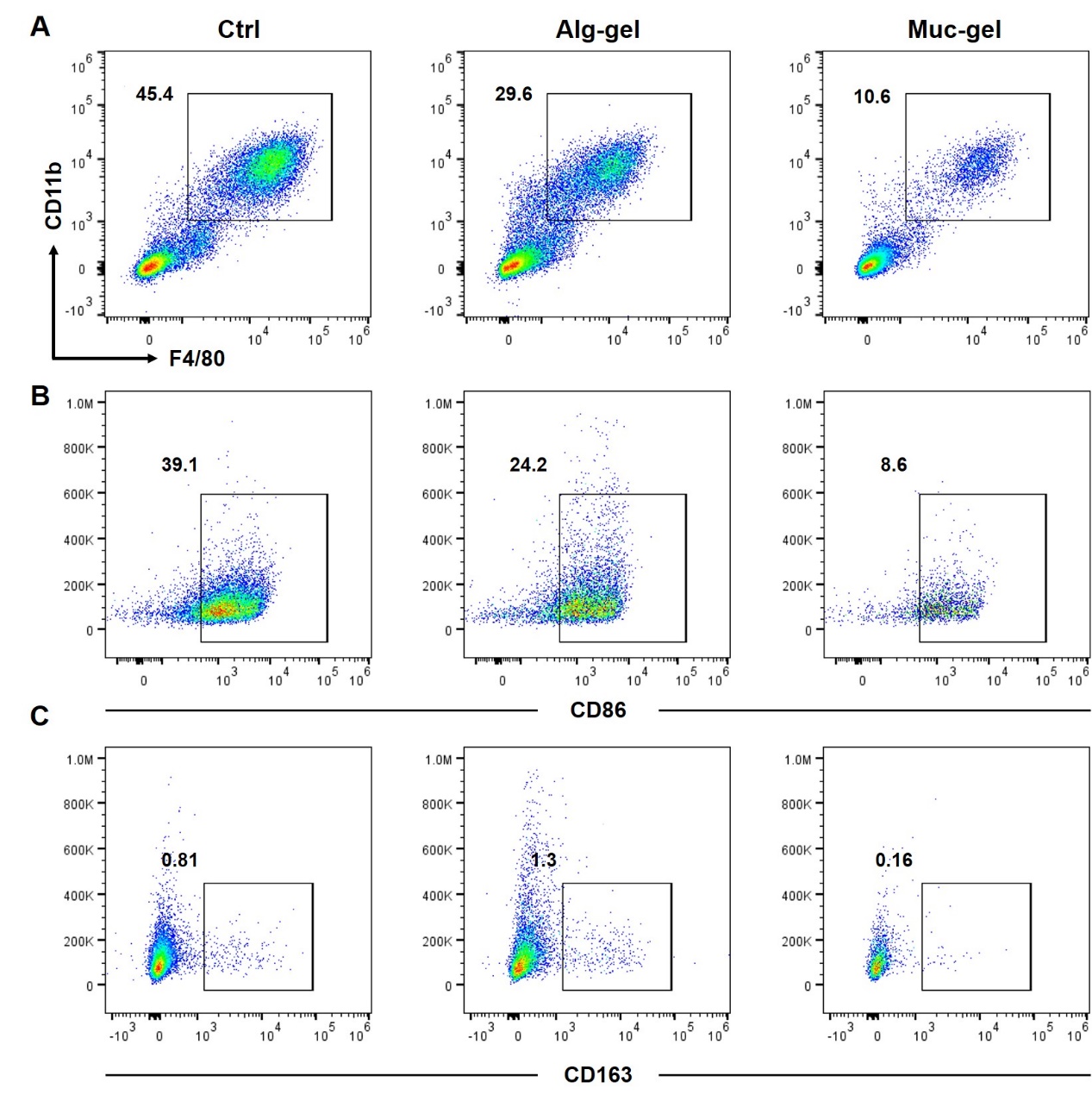


**Fig. S6.** Comparison of macrophage infiltration in gel-encapsulated IVDs and non-encapsulated IVDs in a mouse subcutaneous model and their phenotypic characterization by FACS at week 2. (A-C) Representative FACS graphs of the host macrophages (F4/80^+^ CD11b^+^), M1 macrophages (F4/80^+^ CD11b^+^ CD86^+^), and M2 macrophages (F4/80^+^ CD11b^+^ CD163^+^), respectively. The error bars represent the standard deviation obtained from measurements of n = 9 independent samples from 9 mice, each with three independent repeats.

**Fig. S7**

**
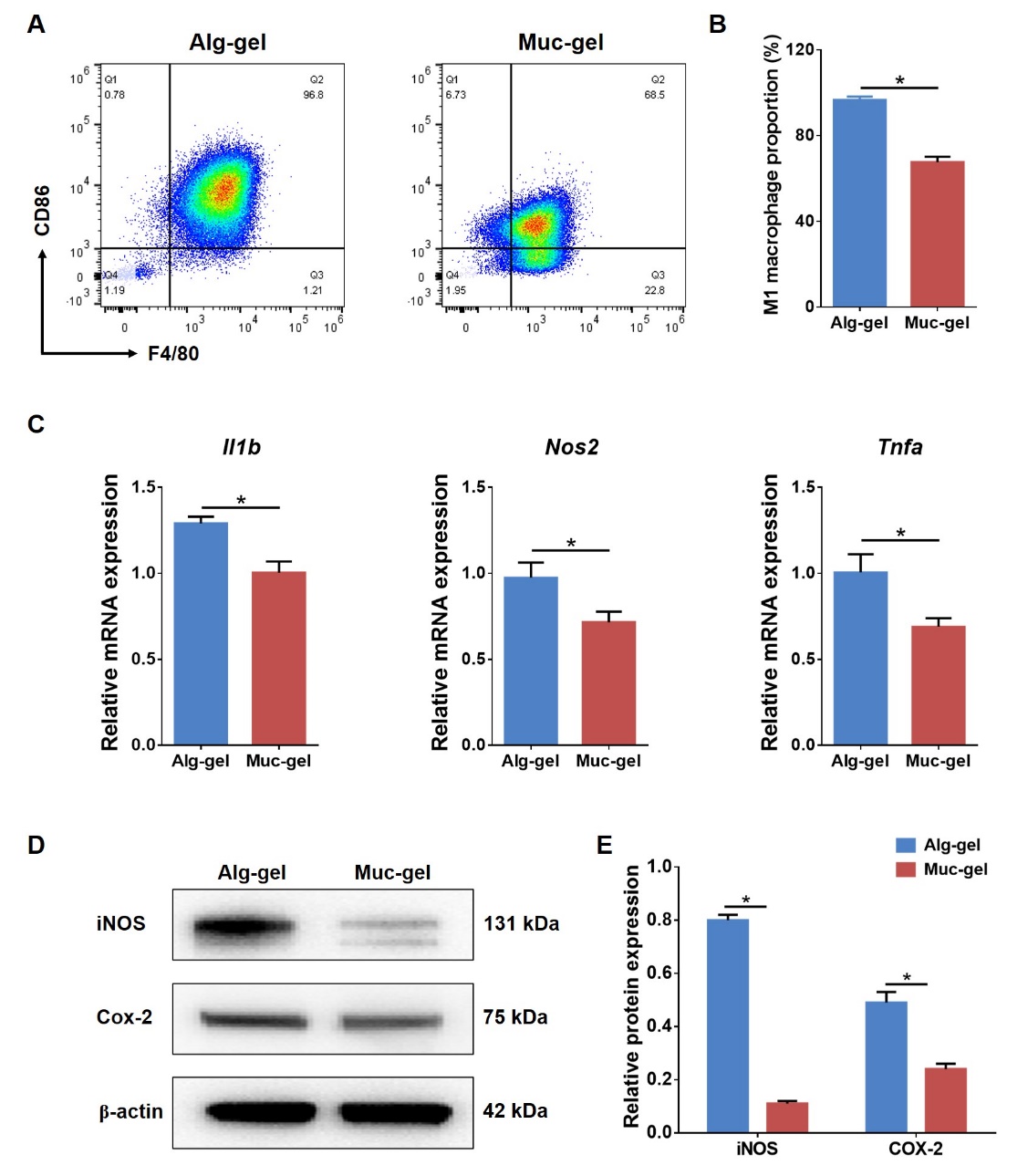
**

**Fig. S7.** Effects of Muc-gels on macrophage polarization *in vitro*. (A) Representative FACS graphs of the M1 macrophages (F4/80^+^ CD86^+^) seeded on Alg-gels and Muc-gels. (B) Proportion of the activation of M1 macrophage within total cell population in the Alg-gel and Muc-gel groups are quantitatively analyzed using Flowjo 10.5.2. (C) qPCR analyses of the relative expression of proinflammatory genes (*Il1b*, *Nos2*, and *Tnfa*) in RAW 264.7 cells of Alg-gel and Muc-gel groups. (D) Western blot analyses of iNOS, Cox-2, and β-actin in RAW 264.7 cells from Alg-gel and Muc-gel groups. (E) Quantification of the relative protein levels of iNOS and Cox-2. The error bars represent the standard deviation obtained from measurements of three independent repeats in each group. Statistical analysis was determined by Prism 9.0 using the unpaired parametric Student’s t-test. *, *p* < 0.05.

**Fig. S8**

**
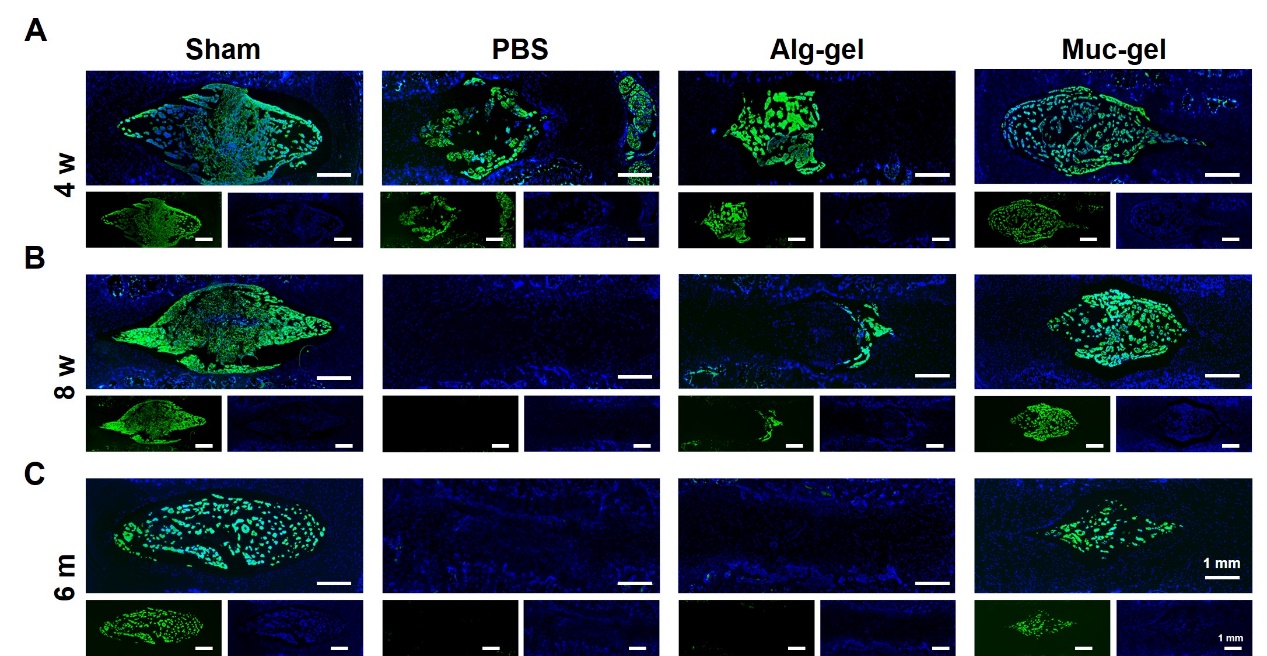
**

**Fig. S8.** Immunofluorescent staining illustrates the expression and spatial distribution of CAXII in IVDs of the Sham, PBS, Alg-gel, and Muc-gel groups at 4 weeks, 8 weeks and 6 months.

**Fig. S9**

**
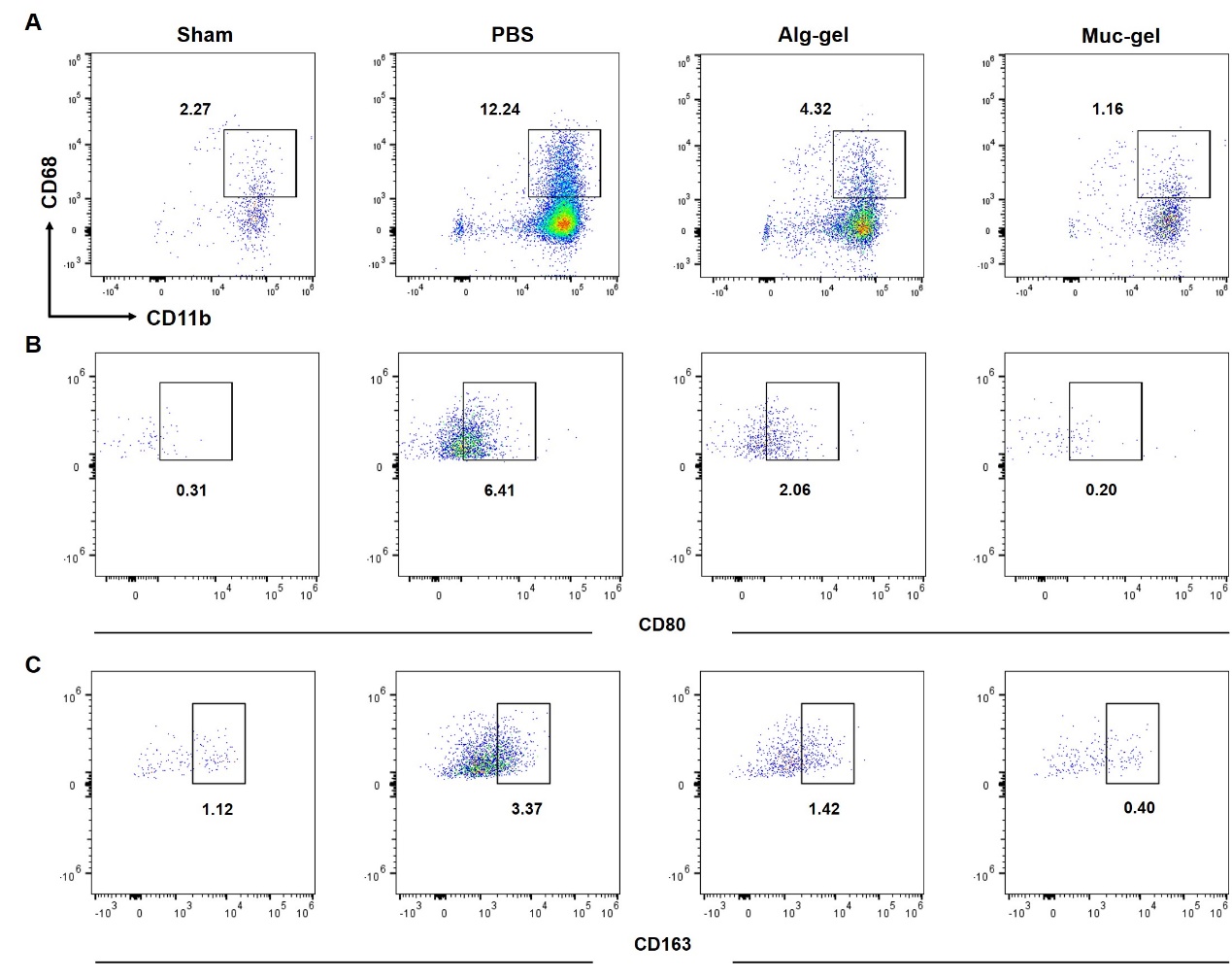
**

**Fig. S9.** Comparison of macrophage infiltration in immediate interventions with Muc-gels in a rat tail IVD degeneration model, alongside Alg-gel, PBS and sham groups and their phenotypic characterization by FACS at week 1. (A-C) Representative FACS graphs of the host macrophages (CD68^+^ CD11b^+^), M1 macrophages (CD68^+^ CD11b^+^ CD80^+^) and M2 macrophages (CD68^+^ CD11b^+^ CD163^+^), respectively.

**Fig. S10**

**
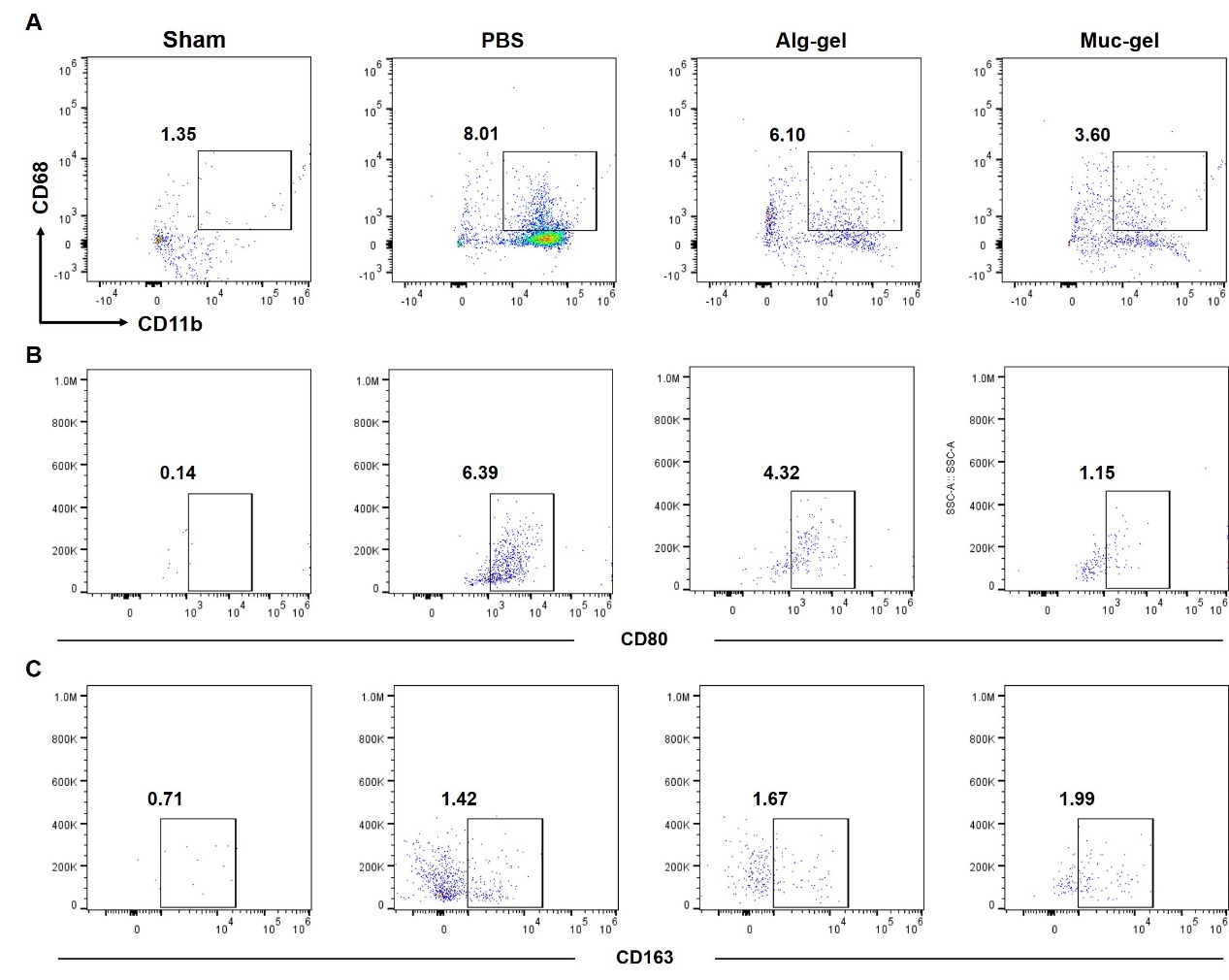
**

**Fig. S10.** Comparison of macrophage infiltration in immediate interventions with Muc-gels in a rat tail IVD degeneration model, alongside Alg-gel, PBS and sham groups and their phenotypic characterization by FACS at week 4. (A-C) Representative FACS graphs of the host macrophages (CD68^+^ CD11b^+^), M1 macrophages (CD68^+^ CD11b^+^ CD80^+^) and M2 macrophages (CD68^+^ CD11b^+^ CD163^+^), respectively.

**Fig. S11**

**
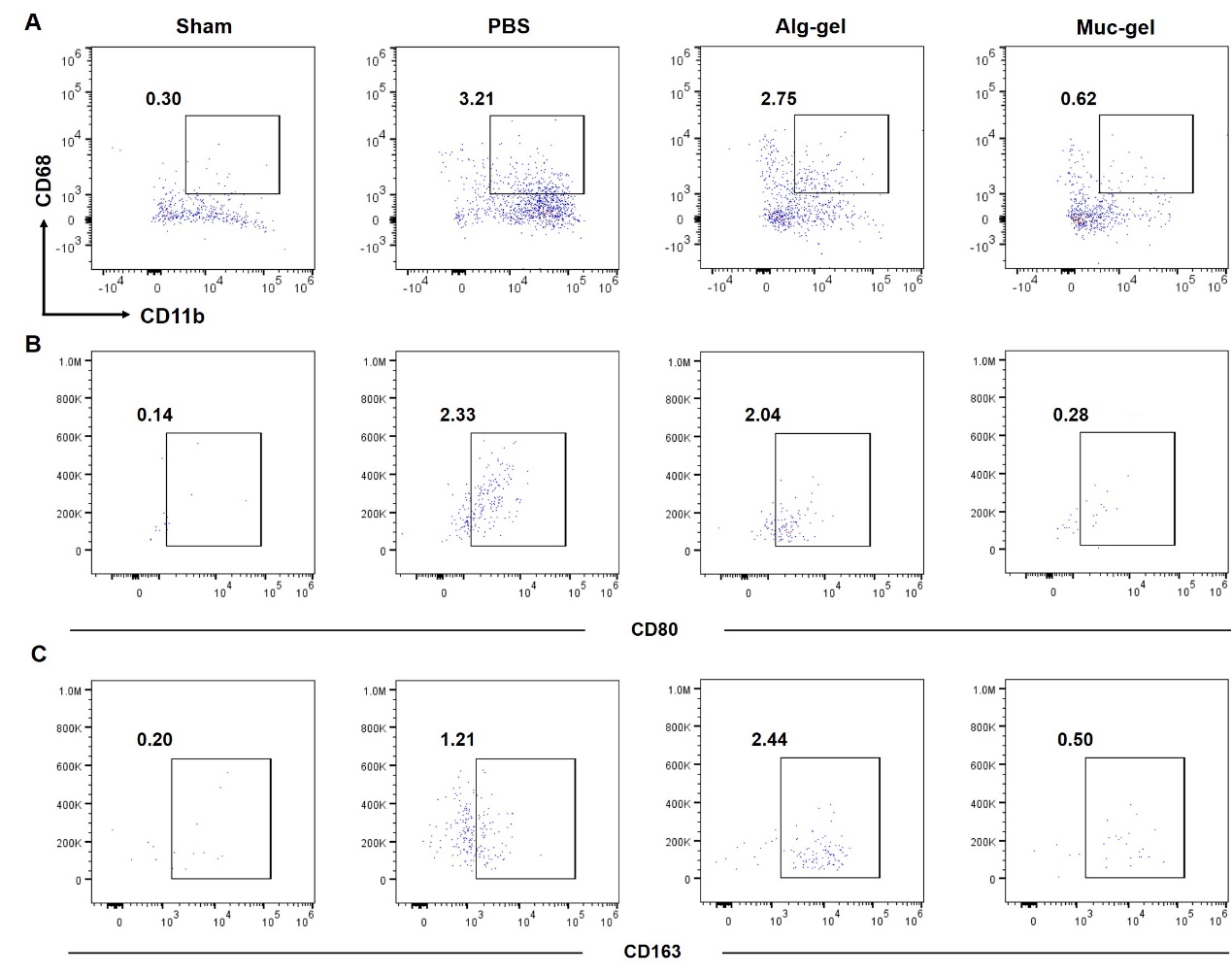
**

**Fig. S11.** Comparison of macrophage infiltration in immediate interventions with Muc-gels in a rat tail IVD degeneration model, alongside Alg-gel, PBS and sham groups and their phenotypic characterization by FACS at week 8. (A-C) Representative FACS graphs of the host macrophages (CD68^+^ CD11b^+^), M1 macrophages (CD68^+^ CD11b^+^ CD80^+^) and M2 macrophages (CD68^+^ CD11b^+^ CD163^+^), respectively.

**Fig. S12**

**
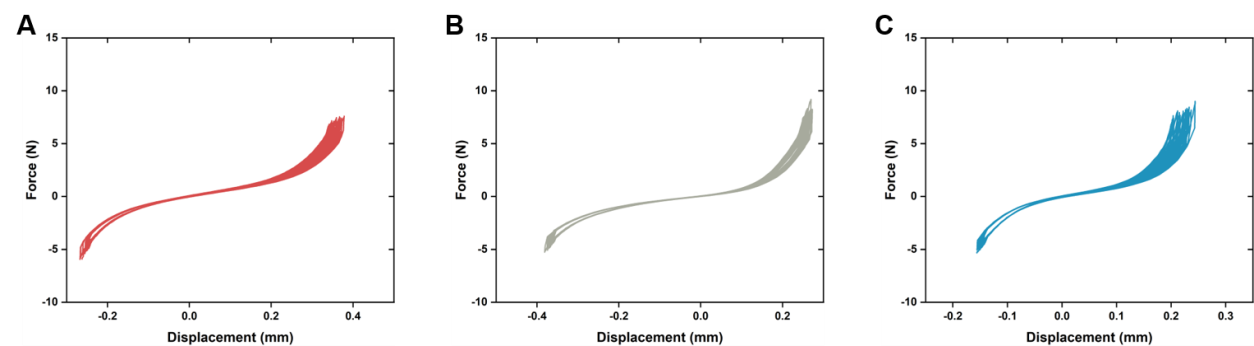
**

**Fig. S12.** Biomechanical analysis of isolated caudal vertebra at 8 weeks after surgery. Representative force-displacement curve of PBS (A), Alg-gel (B), and Muc-gel (C) groups, respectively.

**Fig. S13**


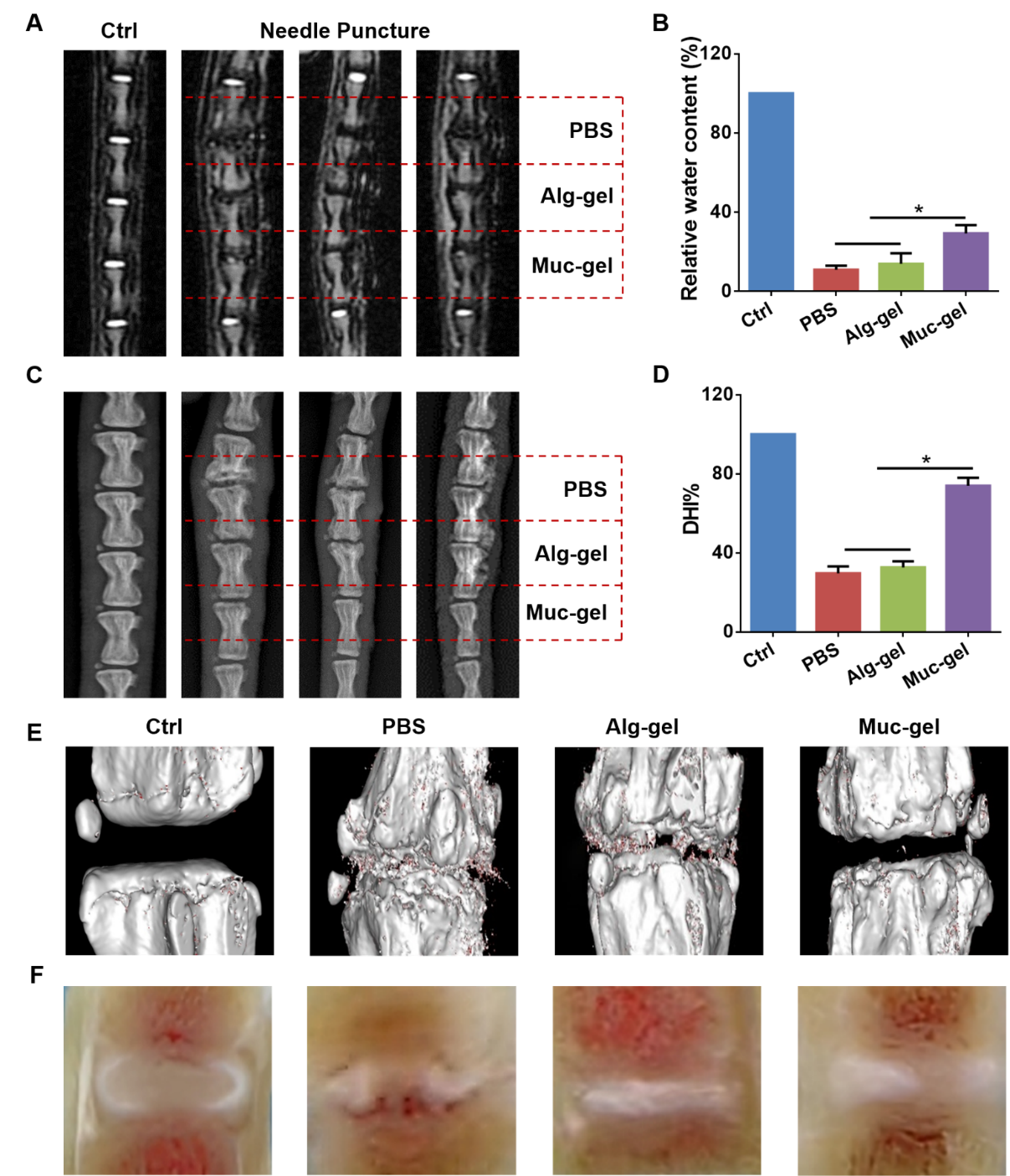


**Fig. S13.** Comparison of immediate interventions with Muc-gels in a rat tail IVD degeneration model with Alg-gels as well as PBS and sham groups at month 6. MRI imaging illustrates the relative water content (A) and disc height index (DHI, C) of IVDs, respectively, along with their quantification (B, D). Micro-CT scanning and gross morphologies provided morphological images of the vertebrae and IVDs respectively (E, F). The error bars represent the standard deviation obtained from measurements of n = 12 independent samples from 12 rats, each with three independent repeats. Statistical analysis was performed using ordinary one-way ANOVA tests with Prima 9.0. *, *p* < 0.05.

**Fig. S14**


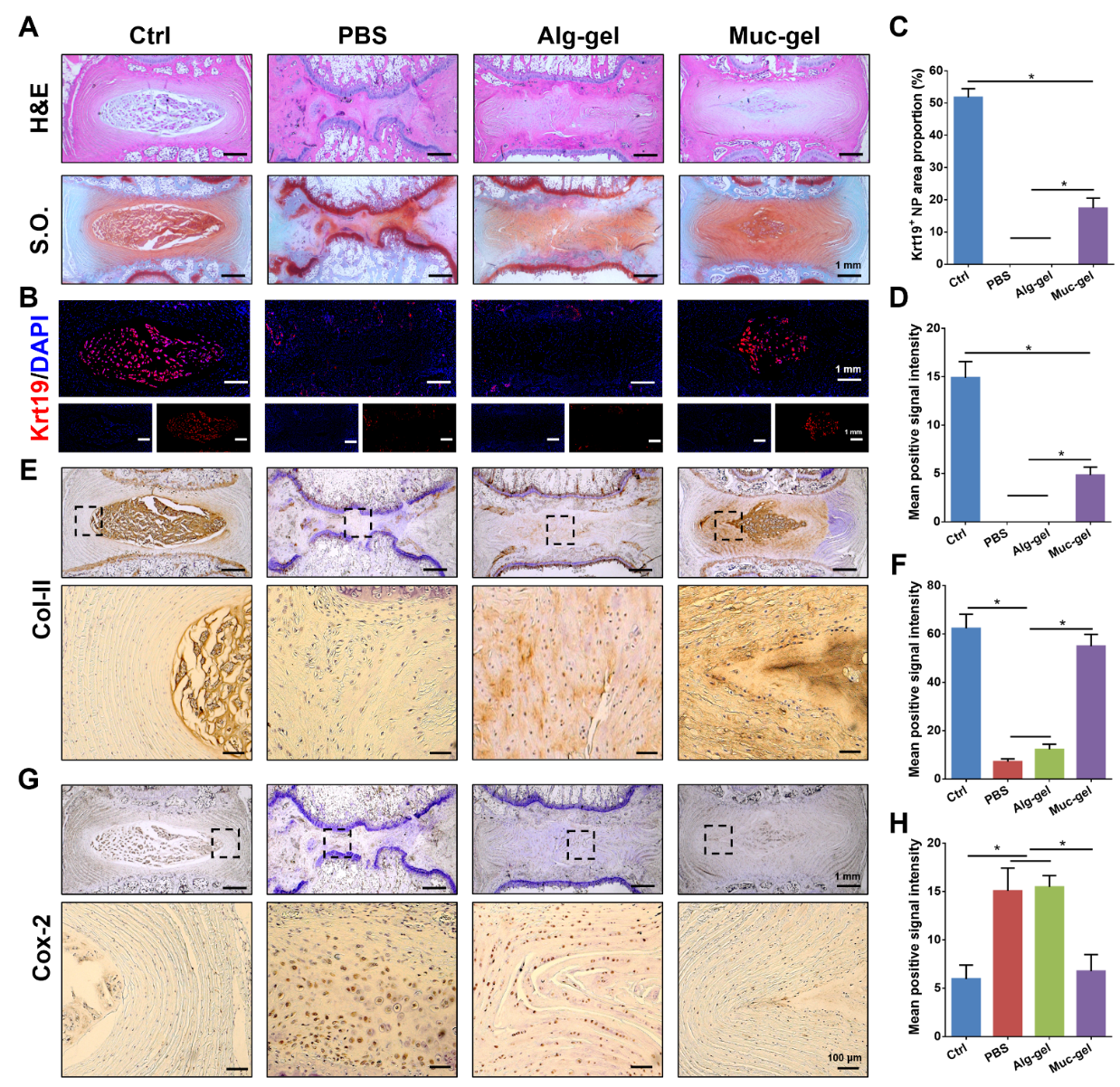


**Figure S14.** Comparison of immediate interventions with Muc-gels in a rat tail IVD degeneration model, alongside Alg-gel, PBS, and sham groups at month 6, using histological, immunofluorescent and immunohistochemistry staining. Hematoxylin and Eosin (H&E) staining and Safranin O- fast green (S.O.) staining illustrate the histological features of IVDs (A). Immunofluorescent staining illustrates the expression and spatial distribution of Krt19, along with their quantification (B-D), using Image J. Immunohistochemistry staining illustrates the express of Col-II and Cox-2 of IVDs (E, G) along with their quantification (F, H), using Image J, respectively. The error bars represent the standard deviation obtained from measurements of n = 12 independent samples from 12 rats, each with three independent repeats. Negative control is shown in **Fig. S15**. Statistical analysis was performed using ordinary one-way ANOVA tests with Prism 9.0. *, *p* < 0.05.

**Fig. S15**

**
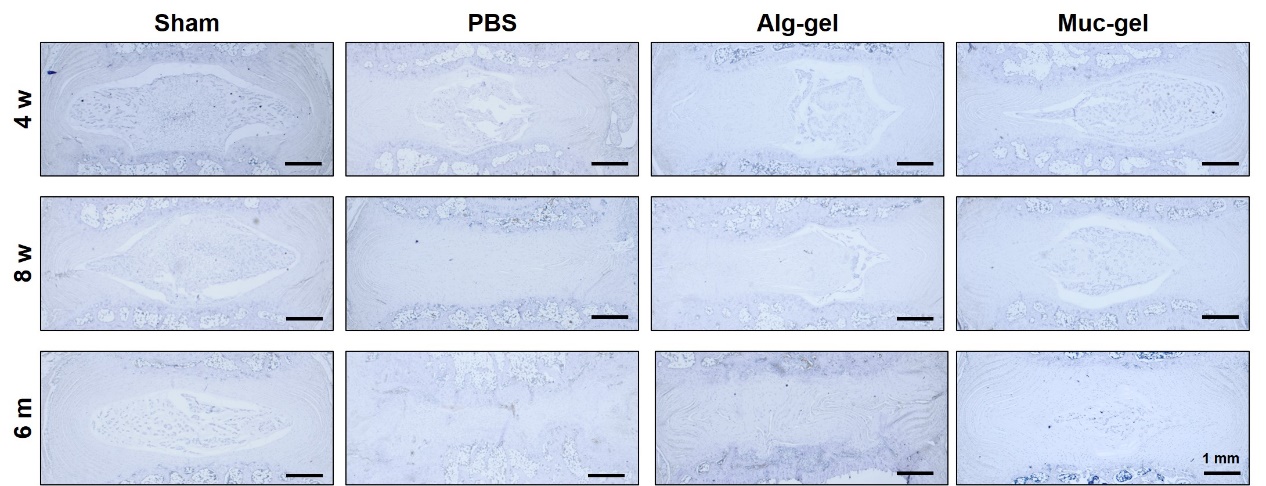
**

**Fig. S15.** Negative control of immunohistochemistry for each group in a rat tail IVD degeneration model at 4 weeks, 8 weeks and 6 months.

**Fig. S16**


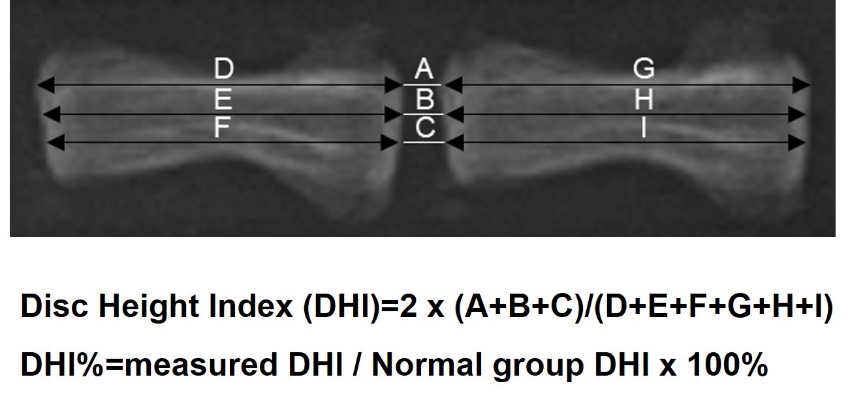


**Fig. S16.** The method of calculating disc height index.
